# Supplementary material for: Physiologically based cord clamping for infants ≥32+0 weeks gestation: A randomised clinical trial and reference percentiles for heart rate and oxygen saturation for infants ≥35+0 weeks gestation
Source: PLoS Med. 2022 Jun 23;19(6):e1004029. doi: 10.1371/journal.pmed.1004029 (PMC9269938; doi:10.1371/journal.pmed.1004029)
Supplement: S2 Supporting information — (PDF) [file pmed.1004029.s002.pdf]

The Baby-Directed Umbilical Cord Cutting (Baby-DUCC) Trial

Statistical Analysis Plan

Table of Contents

Section 1: Administrative information 4
1a. Trial title 4
1b. Trial registration 4
2. SAP version 4
3. Protocol version 4
4. SAP revisions 4
5. Roles and responsibilities 4
Principal Investigators 4
Chief Investigators 4
6. Signatures 5
Section 2: Introduction 6
7. Background and rationale for trial 6
8. Objectives 6
Section 3: Study Methods 7
9. Trial design 7
Study protocol development and conduct 7
Outcomes 7
10. Randomisation 7
11. Sample size 8
12. Framework 8
13. Statistical interim analysis and stopping guidance 8
14. Timing of final analysis 9
Data collection and management 9
15. Timing of outcome assessments 10
Section 4: Statistical Principles 11
Overall principles 11
17. Description and rationale for any adjustment for multiplicity 11
18. Confidence intervals to be reported 11
19. Adherence and protocol deviations 11
20. Analysis population 11
Section 5: Trial population 11
21. Eligibility criteria 11
22. Participant data 12

23. Compliance and concomitant therapies **12**
24. CONSORT diagram **13**
Section 6: Analysis **14**
26. Baseline Characteristics and Study Outcomes **14**
Baseline characteristics **14**
Primary outcome **14**
Secondary outcomes **14**
27. Analysis methods **16**
General **16**
Baseline infant characteristics **17**
Analyses- primary outcome **17**
Subgroup analyses **17**
Analyses: secondary outcomes **17**
Dichotomous secondary outcomes **17**
Continuous secondary outcomes **18**
28. Missing data **18**
29. Additional analyses **18**
30. Safety Outcomes **18**
31. Statistical software **19**
Table 1: Baseline characteristics **20**
Table 2: Primary Outcome **21**
Table 3: Secondary outcomes **21**
Table 4: Safety outcomes **24**
References **25**

Section 1: Administrative information

1a. Trial title

Resuscitation of infants ≥32^{+0} weeks gestation with the umbilical cord intact for improving physiological transition at birth: The Baby-Directed Umbilical Cord Cutting (Baby-DUCC) Trial

1b. Trial registration

ANZCTR registration number ACTRN12618000621213
https://www.anzctr.org.au/Trial/Registration/TrialReview.aspx?id=374884

2. SAP version

Version 5 (dated 2nd June 2021)

3. Protocol version

This document is based on information contained in the study protocol of the Baby-DUCC RCT Protocol Version 8, dated 13th June 2018

4. SAP revisions

Not applicable

5. Roles and responsibilities

Trial Steering Committee

Principal Investigators

Dr Shiraz Badurdeen – Neonatal Clinical/Research Fellow
Newborn Research Centre, RWH, shiraz.badurdeen@thewomens.org.au

Dr Douglas BLANK – Neonatal Consultant
Monash Newborn, Monash Children's Hospital, douglas.blank@monash.edu

Chief Investigators

Dr Omar KAMLIN
    Neonatal Consultant, RWH, omar.kamlin@thewomens.org.au
Dr Calum ROBERTS
    Neonatal Consultant, Monash Children's Hospital, calum.roberts@monash.edu
A/Professor Susan Jacobs
    Deputy Clinical Director of Neonatal Services, RWH Sue.jacobs@thewomens.org.au
Dr Marta THIO
    Neonatal Consultant, RWH, Marta.ThioLluch@thewomens.org.au
Dr Jennifer DAWSON
    Deputy Director of Newborn Research Centre, RWH, Jennifer.Dawson@thewomens.org.au
Dr Stefan KANE
    Obstetric Consultant, RWH, Stefan.Kane@thewomens.org.au
A/Prof Alicia DENNIS
    Director of Anaesthesiology, RWH, alicia.dennis@thewomens.org.au
A/Prof Susan Donath
    Biostatistics, Murdock Children's Institute, Uni Melbourne, susan.donath@mcri.edu.au
A/Prof Graeme POLGLASE
    Physiologist, Hudson Institute, Monash University, graeme.polglase@monash.edu

Prof Stuart HOOPER
Physiologist, Director of Ritchie Centre, Monash University, stuart.hooper@monash.edu
Professor Peter DAVIS
Director of Newborn Research Centre, RWH, pgd@unimelb.edu.au

6. Signatures

This document outlines the proposed statistical analysis plan for the BabyDUCC trial. It was prepared and approved by the BabyDUCC Trial Steering Committee.

Approval:

Dr Shiraz Badurdeen

Principal Investigator
Date: 2nd June 2021

Dr Douglas Blank
Principal Investigator
Date: 2nd June 2021

Prof Susan Donath
Trial Statistician
Date: 2nd June 2021

Section 2: Introduction

7. Background and rationale for trial

Deferred cord clamping (DCC) is recommended for all vigorous infants. Infants who need resuscitation at birth normally receive early cord clamping to be moved to a warming bed to receive assistance to establish breathing.[1] However, the benefits of DCC may be most crucial for the compromised infant. Experiments using newborn lambs conducted by our research team demonstrate that establishing effective ventilation prior to umbilical cord clamping improves physiological stability compared with cutting the umbilical cord and separating the lambs from the placental circulation prior to establishing ventilation.[2-4] Specifically, lambs receiving ventilation prior to umbilical cord clamping have less bradycardia, less fluctuations in blood pressure, improved systemic oxygenation and cerebral perfusion, and improved temperature stability. However, in humans, improved physiologic stability with DCC has not been proven.

Research question

In infants greater than or equal to 32^{+0} weeks gestational age at birth who require resuscitation at birth, does establishing effective ventilation either via positive pressure ventilation (PPV) or effective spontaneous breathing prior to umbilical cord clamping, versus standard care, immediate cord clamping followed by resuscitation, result in improved physiological stability.

8. Objectives

The primary aim is to compare the average heart rate between 60-120 seconds after birth between study arms. Our secondary aims will evaluate the differences in other physiologic and clinical outcomes in the delivery room.

Section 3: Study Methods

9. Trial design

This is an unblinded (to the intervention), parallel arm, randomised controlled trial conducted at The Royal Women's Hospital (RWH) and Monash Medical Centre (MMC), Australia.

Infants are individually randomised 1:1 within 60s of birth if determined to require resuscitation, to either:

1. **BabyDUCC:** Establishment of ventilation, either via positive pressure ventilation or effective spontaneous breathing, prior to umbilical cord clamping
2. **Standard care**: immediate cord clamping followed by resuscitation

Full explanation of the trial design is included in the trial protocol and the Clinical Trials Registry. For all purposes relating to the study, the time of birth is defined by the emergence of the entire fetus from the body of its mother and is unrelated to the time of umbilical cord clamping.

Study protocol development and conduct

The BabyDUCC trial was registered with the Australian and New Zealand Clinical Trials Registry (ACTRN12618000621213) on 20th April 2018.[5] The trial was approved by the Human Research Ethics Committee of The Royal Women's Hospital, Melbourne, Australia (Study ID: 17/19) on 1st June 2018, and by the Human Research Ethics Committee of Monash Health, Melbourne, Australia (Study ID: HREC/18/MonH/19) on 19th April 2018.

The consent process involves written, prospective consent wherever possible from parents for inclusion of their infant in the study. However, in the event of an emergency birth, the study has approval to use a retrospective consent process at the Royal Women's Hospital study site. The infant may be included in the study, then consent to use the collected data will be sought from the parent or guardian as soon as possible after the procedure. Circumstances that were considered appropriate for retrospective consent include:

- Emergency caesarean sections including "code green" caesarean deliveries
- Unplanned caesarean sections with evidence of fetal distress
- Instrumental vaginal births

This consent process was approved by the Human Research Ethics Committee of The Royal Women's Hospital.

An independent data and safety monitoring board (DSMB) is monitoring the study progress. The trial will be reported according to the Consolidated Standards of Reporting Trials (CONSORT) guidelines.[6]

Outcomes

The primary outcome is the average infant heart rate (HR) between 60-120 seconds after birth**.**

Definitions and secondary outcomes are further outlined in Section 6 and in the trial protocol/registry.

10. Randomisation

Each episode is randomly allocated in a 1:1 ratio to either the intervention (BabyDUCC) arm or standard care, stratified by:

1. Centre
2. Indication for paediatric attendance:

a. Gestational age (32+0 to 35+6 weeks)
b. Emergency birth ≥ 36+0 weeks gestation (as defined above)
c. Non-emergency birth ≥ 36+0 weeks gestation

The randomisation sequence uses random permuted blocks with varying block sizes. To enable rapid randomisation following the decision to commence/continue resuscitation, the randomisation is performed at the cot-side using a smartphone with online access to the Research Electronic Data Capture (REDCap) randomisation tool.[7] Randomisation is web-based, using a password-protected, secure sockets layer (SSL)-encrypted website. The randomisation sequence was developed by an independent statistician at the Murdoch Children's Research Institute, Melbourne, Australia.

The randomisation tool enables randomisation to be completed within seconds of the researcher and/or clinical team's decision to commence resuscitation. This decision must be made within 60 seconds of birth of the infant. The group allocations are unblinded, due to the nature of the intervention.

11. Sample size
Based on previous observational data, we hypothesise that infants needing resuscitation who receive Baby-DUCC will have a mean ± standard deviation (SD) HR of 140 ± 30 beats per minute (BPM) between 60-120 seconds after birth and infants needing resuscitation who receive standard care will have a mean ± SD HR of 120 ± 30 BPM. Accepting a 2-sided alpha<0.05 and 90% power (1-beta), we would need to enrol 49 infants into each arm (N=98). To accommodate a 10% attrition rate for detecting the primary outcome due to monitoring failure (based on the Baby-DUCC feasibility pilot study, usually due to poor contact of the ECG leads on the infant), and rounding up for equal numbers in each stratum, we plan to recruit **total N=120**.

12. Framework
The BabyDUCC trial is investigating the superiority of baby-directed umbilical cord clamping, compared with standard care (cord clamping prior to resuscitation) for the primary outcome. Secondary outcomes will also be compared using a superiority framework.

13. Statistical interim analysis and stopping guidance
An external Data Safety and Monitoring Board (DSMB) monitors the trial and comprises:

- Chairperson: Prof Martin Kluckow (Neonatologist, Royal North Shore Hospital)
- Professor Michael Nicholl (Obstetrician, Clinical Director, Maternal Neonatal and Women's Health Network, NSLHD)
- Dr Anneke Grobler (Independent Statistician, Murdoch Children's Research Institute, Melbourne, Australia)

The terms of reference of the DSMB were outlined in the BabyDUCC trial DSMB charter (version number 6, Version date 13th December 2018) and ratified by the Trial Steering Committee and all members of the DSMB during the first DSMB meeting.

Safety analyses, including of pre-defined significant adverse events (SAEs), were planned and performed after recruitment of:

- **36 infants** (30% total)
- **61 infants** (~51% total)

Following the 2nd review at the request of the DSMB, a further safety analysis was conducted at

- **90 patients** (~75% total).

The following events were prespecified and reported as Serious Adverse Events (SAEs):

Maternal:

- Maternal post-partum haemorrhage ≥1L or the need for blood transfusion
- Maternal critical care admission
- Maternal infection up to 30 days after caesarean birth- defined as maternal re-attendance and commencement of antibiotic therapy (oral or intravenous) by emergency or maternity clinical teams
- Maternal thromboembolic event up to 30 days after caesarean birth defined as maternal re-attendance with positive diagnostic imaging
- Maternal death

Neonatal:

- Intubation in the delivery room
- Chest compressions in the delivery room
- Admissions to Neonatal Intensive Care for infants ≥36 weeks gestation
- First temperature <35.5 degrees Celsius
- Pneumothorax treated with needle aspiration and/or chest tube insertion
- Jaundice treated with exchange transfusion
- Polycythaemia requiring partial exchange transfusion as determined by the clinical team
- Hypoxic ischaemic encephalopathy treated with therapeutic hypothermia
- Significant acquired brain injury (on imaging or clinical diagnosis) as documented by the clinical team
- Neonatal death

As per the DSMB charter, the DSMB could recommend stopping the trial on the basis of safety using clinical judgment informed by statistical comparison of adverse event rates. Accumulating signals of harm would not necessarily require statistically significant differences to warrant an alert and recommendation from the DSMB.

After 61 patients were recruited (51% total), an interim efficacy review was undertaken, comparing the two treatment groups (blinded) for the primary endpoint- average HR between 60-120s. The summary data for each study arm was presented by pseudo-labelled treatment arm (e.g. 'A' and 'B') without performing a statistical comparison. The key to identify the treatment arms was able to be supplied by the independent statistician if requested by the DSMB. The DSMB were able to proceed with a statistical comparison and make a recommendation to cease the trial in the presence of very strong interim evidence of a difference between groups in the primary outcome. After reviewing the data, a statistical comparison for the primary outcome between study arms was not performed, and there was no planned adjustment of the significance level due to interim analysis.

At each time-point (30%, 51%, and 75% recruitment), the DSMB recommended continuation of the trial, with an unchanged protocol.

14. Timing of final analysis
Final analysis will be conducted after data entry is completed and the database cleaned and closed.

Data collection and management
Immediately after birth, a researcher applied electrocardiogram (ECG) leads and a pulse oximetry sensor. A GoPro camera (GoPro, San Mateo, California) was focused on the monitor to record HR

and oxygen saturation data as well as details of respiratory support. Audio recording from the video camera was used to verify accurately events during delivery, for example the timing of umbilical cord clamping, the time to initiate breathing, and the timing of pedicap/neostat colour change.

For the heart rate (primary outcome) and oxygen saturation levels, blinded data extraction is performed by a study investigator who was not in attendance at the birth. The video recording is cropped to include only the monitor screen and is muted to all sound before it is shared with this investigator for data extraction. No other clinical or demographic details are provided. HR and oxygen saturation data are extracted at 10 second intervals from birth until 10 minutes of age. ECG and pulse oximetry readings for HR and oxygen saturation will only be used if QRS complexes and plethysmograph waveforms show good quality signal.

Demographic and clinical data are collected and entered directly into the REDCap database by the study investigator attending the birth immediately after the infant is stabilised. Follow up data is extracted from the medical record and entered directly into the REDCap database.

15. Timing of outcome assessments

The primary outcome is average HR between 60-120 seconds after birth determined by 3-lead ECG. Following study enrolment and acquisition of data, the primary outcome is assessed blinded by a study investigator as described above.

The secondary outcomes are measured during the first 10 minutes after birth, and up to hospital discharge for the pre-defined neonatal and maternal safety outcomes. The maternal safety outcome of infection up to 30 days after caesarean birth is determined by reviewing the medical record >30 days after birth.

Section 4: Statistical Principles

Overall principles

The analysis plan detailed below applies to all the data collected in the BabyDUCC randomised trial. Analysis will start once all primary and secondary outcomes are available, missing data has been sought, the database has been cleaned and locked, and the statistical analysis plan has been signed and dated.

17. Description and rationale for any adjustment for multiplicity

All secondary outcomes will be reported as point estimates with unadjusted 95% confidence intervals only.

18. Confidence intervals to be reported

For all outcomes, 95% confidence intervals will be presented.

19. Adherence and protocol deviations

Details for protocol violations for each study group will be provided in a supplementary table. Protocol violations include:

1. cord clamped too early (did not receive intervention)
2. cord clamped too late (did not receive control)
3. oxytocin given too early
4. equipment failure
5. sterility compromised

20. Analysis population

The analysis population will be created by removing the infants who meet post-randomisation exclusion criteria from the randomised population, as outlined below.

1. Randomised in error prior to birth. For N=2 infants as of 9th May 2021, a member of the clinical team assisting the researcher during set-up for the birth inadvertently pressed the 'randomise' button on the smartphone-based randomisation tool. One infant did not require resuscitation, the other receive vigorous stimulation only. The Trial Steering Committee reached a consensus to exclude these infants as the allocation was revealed prior to eligibility being confirmed in terms of assessment for need for resuscitation after birth. Following these 2 errors, the randomisation process was changed to a 2-step randomise-and-confirm process. No further randomisation errors have occurred to date.
2. Parental withdrawal of prospective consent
3. Parental consent declined in retrospective consent group

Therefore, the primary analysis will be based on intention-to-treat (ITT) including all randomised infants regardless of exposure to the allocated treatment or adherence to the trial protocol, excluding the infants who meet the post-randomisation exclusion criteria described above. A per-protocol analysis is not planned.

Section 5: Trial population

21. Eligibility criteria

Infants greater than or equal to 32 weeks' gestation, with a request for a paediatrician to attend the birth for potential newborn distress, born at the participating centres, are eligible for inclusion.

Exclusion criteria:

-Maternal care team determines that deferring umbilical cord clamping or maternal postpartum oxytocin administration until 2-5 minutes after birth is unsafe for the birthing mother

-Monochorionic twins

-Multiples of >2 fetuses

-Known congenital anomalies compromising cardiorespiratory transition after birth:
- -Congenital diaphragmatic hernia
- -Hydrops fetalis
- -Cyanotic congenital heart defects
- -Airway anomalies that may compromise the ability to provide facemask respiratory support.

Following birth, if the infant is vigorous with adequate respiratory effort and does not need resuscitation, the infant will not be randomised. Such infants participate in a separate cohort study of well infants who receive delayed cord clamping. Infants must be assessed to require resuscitation and be randomised prior to 60 seconds after birth to be included in the primary analysis of the BabyDUCC RCT.

22. Participant data
The CONSORT flow diagram shown below will be used to detail enrolment, randomisation, treatment allocation, follow up and analysis.

23. Compliance and concomitant therapies

Infants who did not receive the allocated intervention will be shown in the 'Allocation' section of the CONSORT diagram along with reasons. Compliance is defined as follows:
- BabyDUCC group: cord clamping occurring ≥ 120 seconds after birth
- Standard care group: cord clamping occurring within 60 seconds after birth.

Neither the provision of resuscitation nor the timing of oxytocin administration are included in the definition of compliance. Details of these concomitant therapies will be shown instead in Tables 1 and 3 (see below).

24. CONSORT diagram

CONSORT 2010 Flow Diagram Outline for BabyDUCC RCT

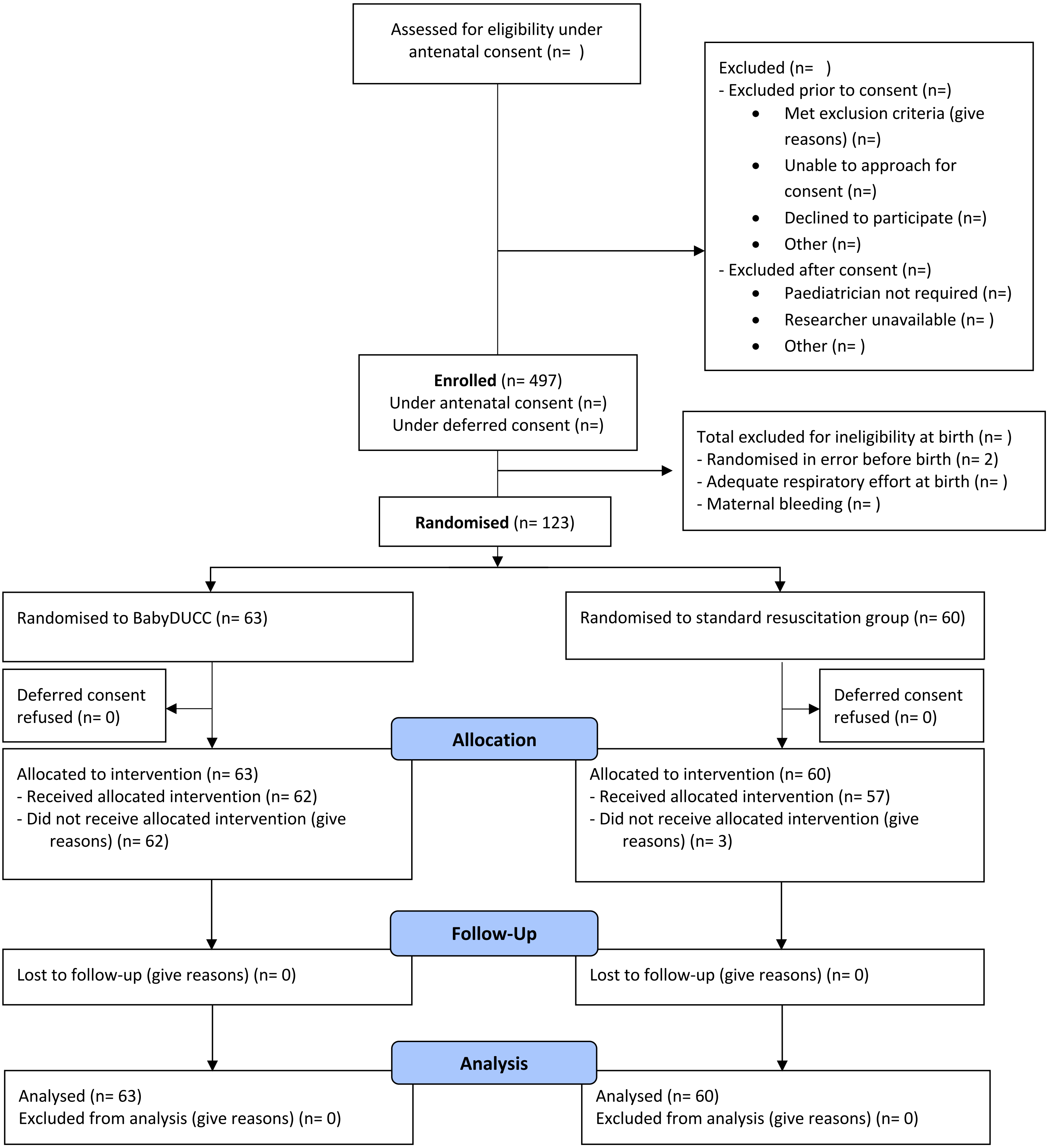

Section 6: Analysis

26. Baseline Characteristics and Study Outcomes

Baseline characteristics

Variables to be reported as baseline characteristics are shown in Table 1 below. Included are the following variables that were registered as secondary outcomes in the study protocol but are more reflective of adherence to study procedures. They will therefore be reported in Table 1:

- Time from birth to umbilical cord clamping
- Time from birth to oxytocin delivery to the mother if administered
- Time from birth to obtain accurate data from ECG

Primary outcome
The primary outcome is average HR between 60-120 seconds after birth determined by 3-lead ECG. For each infant, the average of the HR measurements at 10 second intervals between 60-120 seconds after birth will be calculated (database variable name *AveHR60.120*, which is derived from variables from hr0060 to hr00120).

As per the study protocol, infants included in the primary analysis must have
(i) HR data at 4/7 data points, and
(ii) at least one HR data point by 80 seconds after birth.
The primary outcome will be considered missing for infants without both (i) and (ii).

The primary analysis will be a complete case analysis. If the proportion of missing data for the primary outcome is more than 5%, analysis based on multiple imputation may be performed as detailed in section 28.

Secondary outcomes

Outcome No.	Outcome	Variable in database
**Infant Outcomes**		
1.	Any resuscitation*<br><br>- Stimulation alone<br>- Supplemental oxygen<br>- CPAP (with or without oxygen)<br>- Positive pressure ventilation (mask)<br>- intubation<br>- chest compressions	- respsuppyn, vstimyn<br><br>- vstimyn<br>- typesupp<br>- typesupp<br>- typesupp<br><br>- delintubyn<br>- ccompryn
2.	Time from birth to initiate respiratory support, if the infant received respiratory support	tinsupp

3.	Time from birth to pedicap/neostat colour change, if the infant received respiratory support	tcolourch
4.	Time from birth to 1st cry	tfcry
5.	Time between randomisation and first cry for infants who did not receive respiratory support^	tfcry, trandom
5.	Time to regular respirations	tregresp
6.	Maximum fraction of inspired oxygen	maxo2
7.	Proportion of infants with heart rate <100 beats per minute	Variables from *hr0060* to *hr0600,* representing HR every 10s from 60s to 10 min
8.	Time spent with heart rate < 100 beats per minute	Variables from *hr0060* to *hr0600,* representing HR every 10s from 60s to 10 min
9.	Time spent with heart rate > 180 beats per minute	Variables from *hr0060* to *hr0600,* representing HR every 10s from 60s to 10 min
10.	Heart rate variability	Comparison of the standard deviations of *hr0060* to *hr0600* between groups
11.	Change in heart rate over time in the 10 min after birth	Variables from *hr0060* to *hr0600,* representing HR every 10s from 60s to 10 min
12.	Change in oxygen saturation over time in the 10 min after birth	Variables from *sp0060* to *sp0600,* representing oxygen saturation every 10s from 60 s to 10 min
13.	Apgar score at 1 min	ap1min
14.	Apgar score at 5 min	ap5min
15.	Apgar score at 10 min	ap10min
16.	First temperature	temp
17.	Cord arterial pH	cphart
18.	Cord arterial lactate	clacart
19.	Cord venous pH	cphven
20.	Cord venous lactate	clacven
21.	Proportion admitted primarily for respiratory support	admr
22.	Proportion admitted for other reason<br>- prematurity or low birthweight alone<br>- low glucose<br>- other	admr
23.	Birthweight	wt
24.	Proportion receiving phototherapy	phototx
25.	Proportion of infants treated for polycythaemia	polycytx
26.	Proportion of infants receiving an exchange transfusion	exchtx

27.	Haematocrit level if measured, within 24 h of birth	d1hct
**Maternal outcomes**		
28.	Maternal blood loss in ml#	meblcont
29.	Proportion with postpartum haemorrhage<br>- 500ml – 999ml<br>- >= 1000 ml<br>- received blood transfusion	meblcat<br><br>- meblcat<br>- meblcat<br>- pphtx
30.	Proportion with retained placenta	retplta
31.	Maternal infection<br>- following vaginal birth<br>- up to 30 days after caesarean birth	 - minfvag<br>- minfcs

Additional notes on secondary outcomes

1. The following secondary outcome was specified in our trial protocol:

Rates of successful umbilical cord blood donation as applicable

However, cord blood donation did not occur for infants recruited in the trial. Therefore, we have no data to report on this outcome.

2. (*) The proportion of infants in each group who did not receive any resuscitation will be shown as a footnote to the secondary outcomes table (see Table 3 below) and reported in the text of the Results section. These infants cried immediately after randomisation.

3. During trial recruitment it became apparent that a significant proportion of randomised infants cried without respiratory support. The study intervention is defined as "establishment of ventilation, either via positive pressure ventilation or effective spontaneous breathing, prior to umbilical cord clamping" (section 3, 9.1). *Time between randomisation and first cry for infants who did not receive respiratory support* (^) has therefore been added as a secondary outcome although it was not prespecified as a secondary outcome in the study protocol.

3. *Maternal blood loss in ml* (#) was not prespecified in the protocol. During the trial, the DSMB monitored maternal blood loss closely. We therefore pre-specify here the reporting of the mean (or median) volume of blood loss for mothers in each study group.

27. Analysis methods

General

Details of the randomisation will be unblinded only once the sections of the database containing the baseline variables and primary and secondary outcome variables for the RCT has been locked and the Statistical Analysis Plan has been finalised and approved by the trial team.

Statistical analyses will follow standard methods for randomised controlled trials and the primary analysis will be by intention to treat (ITT), including all randomised participants where outcome data are available.

Baseline infant characteristics

The baseline characteristics of the infants will be summarised by group. Binary and categorical variables will be presented as the number and proportion in each category. Continuous variables will be presented as means and standard deviations (SDs), or medians and interquartile ranges (IQR) for skewed data. The list of baseline characteristics that will be summarised is shown in Table 1.

Analyses- primary outcome

The distribution of the primary outcome will be assessed visually using graphical methods (histogram and dotplot). If the distribution is considered to be so skewed that the mean is an inappropriate summary measure, the primary outcome will be summarised using the median, otherwise the primary outcome will be summarized by the mean.

If the summary measure is the mean, the mean and SD HR will be presented separately for the 2 treatment groups. Comparison between the treatment arms will be estimated using linear regression adjusted for the stratification factors used during randomisation. Results will be presented as difference of means with 95% confidence interval (CI) and p value from the regression.

If the summary measure is the median, the median and IQR HR will be presented separately for the 2 treatment groups. Comparison between the treatment arms will be estimated using quantile regression adjusted for the stratification factors used during randomisation. Results will be presented as difference of medians with 95% confidence interval (CI) and p value from the regression.

Subgroup analyses

As the study is not powered for subgroup analysis, these analyses are considered exploratory. Two additional adjusted models will be estimated to explore potential heterogeneity of the effect of the intervention. Each model will include as covariates the stratification factors used in randomisation, and an interaction term estimating the interaction between the intervention and the subgroup variable (listed below). Specific subgroup estimates and confidence intervals will be presented obtained from the adjusted model. If there is no evidence of interaction (p>0.05), any differences between subgroups will be regarded as due to chance.

Subgroup description	Variable in database
Indication for paediatric attendance<br>• Gestational age (32+0 to 35+6 weeks)<br>• Emergency birth ≥ 36+0 weeks gestation (as defined above)<br>• Non-emergency birth ≥ 36+0 weeks gestation	 • gestagedec<br>• delmode; gestagedec<br><br>• delmode; gestagedec

Analyses: secondary outcomes

Dichotomous secondary outcomes

For each dichotomous secondary outcome, the percentage of study participants with the outcome will be presented separately for infants in the treatment and non-treatment groups. Comparison between the treatment arms will be estimated using binary regression adjusted for the stratification

factors used during randomisation. Results will be presented as risk difference and its 95% confidence interval (CI). Dichotomous secondary outcomes are listed in Table 3.

Continuous secondary outcomes

The distribution of each secondary continuous outcome will be assessed visually using graphical methods (histogram and dotplot). If the distribution is considered to be so skewed that the mean is an inappropriate summary measure, the outcome will be summarised using the median, otherwise the outcome will be summarized by the mean.

If the summary measure is the mean, the mean and SD HR will be presented separately for the 2 treatment groups. Comparison between the treatment arms will be estimated using linear regression adjusted for the stratification factors used during randomisation. Results will be presented as difference of means with 95% confidence interval (CI).

If the summary measure is the median, the median and IQR HR will be presented separately for the 2 treatment groups. Comparison between the treatment arms will be estimated using quantile regression adjusted for the stratification factors used during randomisation. Results will be presented as difference of medians with 95% confidence interval (CI).

Continuous secondary outcomes that will be analysed in this way are listed in Table 3.

For continuous secondary outcomes comparing differences between randomised groups over time, the results will be presented graphically with the mean parameter value at each timepoint for each treatment group +/- 95% confidence interval.

Continuous secondary outcomes that will be analysed in this way include the following:

1. Change in HR over time
2. Change in oxygen saturation over time

28. Missing data

Every attempt will be undertaken to retrieve missing data. The primary analysis will be a complete case analysis. If the proportion of missing data on the primary outcome is more than 5%, the rate and patterns of missing data will be examined and, if appropriate, a sensitivity analysis will be performed to compare the results of analyses restricted to infants with complete data and analyses where those with missing data are included using multiple imputation. If used, multiple imputation models will be conducted for the outcome variables and 50 completed data sets will be imputed by chained equations including all the infants initially randomised. The primary outcome, strata variables (centre and indication for paediatric attendance:), and variables predictive of (i) missingness and /or (ii) HR will be included in the imputation model.

29. Additional analyses

Subsequent analyses that are not specified in the protocol may be performed if requested by journal editors or reviewers. These will be performed consistently with the principles of this analysis plan, as far as possible. Subsequent analyses of a more exploratory nature will not be bound by this strategy but are expected to follow the broad principles described.

30. Safety Outcomes

The incidence of each of the Serious Adverse Events listed in section 13 will be compared between groups. In addition, the incidence of the following Adverse Events will be reported for each group:

- Maternal post-partum haemorrhage between 500ml-999ml without blood transfusion
- First temperature <36.5 degrees Celsius

Safety outcomes will be presented as shown in Table 4.

31. Statistical software
Data will be analysed using STATA, *R* and PRISM.

Table 1: Baseline characteristics

	**Variable(s) in database**	**BabyDUCC group (N = XX)**	**Control group (N = XX)**
Hospital at birth<br>- Royal Women's Hospital<br>- Monash Medical Centre	redcap_data_access_group	 XX (%)<br>XX (%)	 XX (%)<br>XX (%)
Antenatal consent	consenttype	XX (%)	XX (%)
**Maternal**			
Maternal age (years)	mage	Median (IQR)	Median (IQR)
Primiparity	primipyn	XX (%)	XX (%)
Any medical complication of pregnancy[a]	matconditions	XX (%)	XX (%)
Spontaneous onset of labour	dindication_spont	XX (%)	XX (%)
Antenatal oxytocin infusion	syntyn	XX (%)	XX (%)
Strongest analgesia<br>- None/Nitrous oxide<br>- Opiate (IM/IV)<br>- Epidural/Spinal<br>- General anaesthetic	anaesth	XX (%)	XX (%)
**Birth**			
Reason for paediatric attendance<br>- Preterm<br>- Fetal growth restriction<br>- Meconium-stained amniotic fluid<br>- Abnormal CTG<br>- Breech/transverse lie<br>- Instrumental birth<br>- Unplanned caesarean section	 - gestagedec<br>- dindication_iugr<br>- comprotype_mec<br>- comprotype_abnctg<br>- dindication_breech<br>- delmode<br>- delmode	XX (%)	XX (%)
Labour complications<br>- Failure to progress<br>- Prolonged 2nd stage<br>- Difficult extraction<br>- None	 - delcompli_ftp<br>- delcompli_pr2st<br>- delcompli_extrac<br>- delcompli_none	XX (%)	XX (%)
Last fetal heart rate	fhr	Median (IQR)	Median (IQR)
Gestational age (weeks^{+days})	gestagedec	Median (IQR)	Median (IQR)
Birthweight (kg)	wt	Median (IQR)	Median (IQR)
Female sex	gender	XX (%)	XX (%)
Time from birth to<br>- randomisation<br>- cord clamping (s)<br>- maternal oxytocin administration	 - trandom<br>- tucc<br>- toxy	Mean (SD)	Mean (SD)

Time from birth to obtain accurate data from the ECG or pulse oximeter	thrdata	Mean (SD)	Mean (SD)

[a] Includes hypertensive disorders of pregnancy, diabetes mellitus, sepsis, oligohydramnios, antepartum haemorrhage, placenta previa

Table 2: Primary Outcome

	BabyDUCC group, n = XX N(%)	Control group, n = XX N(%)	Difference of means (BabyDUCC minus Control) (95% CI)	p-value
**Primary Outcome**				
Mean infant HR between 60-120 seconds after birth	Mean (SD)	Mean (SD)	Mean difference	
Mean infant HR between 60-120 seconds after birth for each subgroup:				
- 32+0 to 35+6 weeks' gestation	Mean (SD)	Mean (SD)	Mean difference	N/A*
- Emergency birth ≥ 36+0 weeks gestation	Mean (SD)	Mean (SD)	Mean difference	N/A*
- Non-emergency birth ≥ 36+0 weeks gestation	Mean (SD)	Mean (SD)	Mean difference	N/A*

* Will be included if p value for interaction < 0.05

Table 3: Secondary outcomes

**Binary outcomes**	BabyDUCC group, n = XX N(%)	Control group, n = XX N(%)	Risk Difference (95% CI)
**Resuscitation**			
Any resuscitation in the delivery room*	XX (%)	XX (%)	XX (%)
- Stimulation alone	XX (%)	XX (%)	
- Supplemental oxygen	XX (%)	XX (%)	
- CPAP (with or without oxygen)	XX (%)	XX (%)	
- Positive pressure ventilation (mask)	XX (%)	XX (%)	
- Intubation	XX (%)	XX (%)	
- Chest compressions	XX (%)	XX (%)	
Proportion of infants with heart rate <100 beats per minute	XX (%)	XX (%)	XX (%)

**Infant**			
Proportion admitted primarily for respiratory support	XX (%)	XX (%)	XX (%)
Proportion admitted for other reason<br><br>- prematurity or low birthweight alone<br>- low glucose<br>- other	XX (%)<br><br>XX (%)<br>XX (%)<br>XX (%)	XX (%)<br><br>XX (%)<br>XX (%)<br>XX (%)	XX (%)
Proportion receiving phototherapy	XX (%)	XX (%)	XX (%)
Proportion treated for polycythaemia	XX (%)	XX (%)	XX (%)
Proportion receiving an exchange transfusion	XX (%)	XX (%)	XX (%)
**Maternal**			
Proportion with postpartum haemorrhage<br><br>- 500ml – 999ml<br>- >= 1000 ml<br>- received blood transfusion	XX (%)<br><br>XX (%)<br>XX (%)<br>XX (%)	XX (%)<br><br>XX (%)<br>XX (%)<br>XX (%)	XX (%)
Proportion with retained placenta	XX (%)	XX (%)	XX (%)
Maternal infection<br><br>- following vaginal birth<br>- up to 30 days after caesarean birth	XX (%)<br><br>XX (%)<br>XX (%)	XX (%)<br><br>XX (%)<br>XX (%)	XX (%)
**Continuous outcomes[#]**	**BabyDUCC group, n = XX Mean (SD)**	**Control group, n = XX Mean (SD)**	**Mean Difference (95% CI)**
**Resuscitation**			
Time from birth to initiate respiratory support (s)	Mean (SD)	Mean (SD)	Mean Difference
Time from birth to pedicap colour change (s)	Mean (SD)	Mean (SD)	Mean Difference
Time from birth to first cry (s)	Mean (SD)	Mean (SD)	Mean Difference
Infants who did not receive respiratory support: Time between randomisation and first cry	Mean (SD)	Mean (SD)	Mean Difference
Time to regular respirations (s)	Mean (SD)	Mean (SD)	Mean Difference
Maximum fraction of inspired oxygen	Mean (SD)	Mean (SD)	Mean Difference
Time spent with heart rate < 100 beats per minute (s)	Mean (SD)	Mean (SD)	Mean Difference
Time spent with heart rate > 180 beats per minute (s)	Mean (SD)	Mean (SD)	Mean Difference
Heart rate variability (bpm)	Mean (SD)	Mean (SD)	Mean Difference
Apgar score at 1 min	Mean (SD)	Mean (SD)	Mean Difference
Apgar score at 5 min	Mean (SD)	Mean (SD)	Mean Difference

Apgar score at 10 min	Mean (SD)	Mean (SD)	Mean Difference
First temperature (C)	Mean (SD)	Mean (SD)	Mean Difference
Cord arterial pH	Mean (SD)	Mean (SD)	Mean Difference
Cord arterial lactate	Mean (SD)	Mean (SD)	Mean Difference
Cord venous pH	Mean (SD)	Mean (SD)	Mean Difference
Cord venous lactate	Mean (SD)	Mean (SD)	Mean Difference
**Infant**			
Birthweight (g)	Mean (SD)	Mean (SD)	Mean Difference
Haematocrit level if measured, within 24 h of birth	Mean (SD)	Mean (SD)	Mean Difference
**Maternal**			
Maternal blood loss (ml)	Mean (SD)	Mean (SD)	Mean Difference

* The proportion of infants in each group who did not receive any resuscitation will be shown as a footnote to the table. These infants cried immediately after randomisation.

[#] Continuous outcomes that are skewed will be reported as median (IQR)

Bpm- beats per minute

Table 4: Safety outcomes

	Variable(s) in database	BabyDUCC group (N = XX)	Control group (N = XX)	Risk Difference (95% CI)
**Maternal**				
Post-partum haemorrhage ≥1L or the need for blood transfusion	meblcat, pphtx	XX (%)	XX (%)	XX (%)
Maternal post-partum haemorrhage between 500ml-999ml without blood transfusion	meblcat, pphtx	XX (%)	XX (%)	XX (%)
Critical care admission	matccuyn	XX (%)	XX (%)	XX (%)
Infection up to 30 days after caesarean birth^	minfcs, minfvag	XX (%)	XX (%)	XX (%)
Thromboembolic event up to 30 days after caesarean birth	mothersaetype	XX (%)	XX (%)	XX (%)
Maternal death	matdeathyn	XX (%)	XX (%)	XX (%)
**Infant**				
Intubation in the delivery room	delintubyn	XX (%)	XX (%)	XX (%)
Chest compressions in the delivery room	ccompryn	XX (%)	XX (%)	XX (%)
Admissions to Neonatal Intensive Care for infants ≥36 weeks gestation	bsaetype	XX (%)	XX (%)	XX (%)
First temperature <36.5 degrees Celsius	temp	XX (%)	XX (%)	XX (%)
First temperature <35.5 degrees Celsius	temp	XX (%)	XX (%)	XX (%)
Pneumothorax treated with needle aspiration and/or chest tube insertion	bsaetype	XX (%)	XX (%)	XX (%)
Jaundice treated with exchange transfusion	bsaetype	XX (%)	XX (%)	XX (%)
Polycythaemia requiring partial exchange transfusion	bsaetype	XX (%)	XX (%)	XX (%)
Hypoxic ischaemic encephalopathy treated with therapeutic hypothermia	bsaetype	XX (%)	XX (%)	XX (%)
Significant acquired brain injury (on imaging or clinical diagnosis) as documented by the clinical team	bsaetype	XX (%)	XX (%)	XX (%)
Neonatal death	bsaetype	XX (%)	XX (%)	XX (%)

^ Maternal post-caesarean infection was defined as maternal hospital re-attendance and commencement of antibiotic therapy (oral or intravenous) by emergency or maternity clinical teams

References

1. Wyckoff MH, Aziz K, Escobedo MB, Kapadia VS, Kattwinkel J, Perlman JM, Simon WM, Weiner GM, Zaichkin JG: **Part 13: Neonatal Resuscitation: 2015 American Heart Association Guidelines Update for Cardiopulmonary Resuscitation and Emergency Cardiovascular Care**. *Circulation* 2015, **132**(18 Suppl 2):S543-560.
2. Bhatt S, Alison BJ, Wallace EM, Crossley KJ, Gill AW, Kluckow M, te Pas AB, Morley CJ, Polglase GR, Hooper SB: **Delaying cord clamping until ventilation onset improves cardiovascular function at birth in preterm lambs**. *The Journal of physiology* 2013, **591**(Pt 8):2113-2126.
3. Polglase GR, Dawson JA, Kluckow M, Gill AW, Davis PG, Te Pas AB, Crossley KJ, McDougall A, Wallace EM, Hooper SB: **Ventilation onset prior to umbilical cord clamping (physiological-based cord clamping) improves systemic and cerebral oxygenation in preterm lambs**. *PLoS One* 2015, **10**(2):e0117504.
4. Blank DA, Crossley KJ, Kashyap AJ, Hodges RJ, DeKoninck PLJ, McGillick EV, Rodgers KA, te Pas AB, Hooper SB, Polglase GR: **Physiologic-Based Cord Clamping Maintains Core Temperature vs. Immediate Cord Clamping in Near-Term Lambs**. *Frontiers in Pediatrics* 2020, **8**.
5. ANZCTR - Registration. https://www.anzctr.org.au/Trial/Registration/TrialReview.aspx?id=374884. Accessed November 20, 2019.
6. Harris PA, Taylor R, Minor BL, et al. The REDCap consortium: **Building an international community of software platform partners.** J Biomed Inform. 2019;95. doi:10.1016/j.jbi.2019.103208
7. Schulz KF, Altman DG, Moher D, Group C: **CONSORT 2010 statement: updated guidelines for reporting parallel group randomised trials**. *BMJ* 2010, **340**:c332.
