# Supplementary material for: Physiologically based cord clamping for infants ≥32+0 weeks gestation: A randomised clinical trial and reference percentiles for heart rate and oxygen saturation for infants ≥35+0 weeks gestation
Source: PLoS Med. 2022 Jun 23;19(6):e1004029. doi: 10.1371/journal.pmed.1004029 (PMC9269938; doi:10.1371/journal.pmed.1004029)
Supplement: S3 Supporting information — (PDF) [file pmed.1004029.s003.pdf]

The Baby-Directed Umbilical Cord Cutting Physiology Study: A Randomised Controlled Trial

Table of Contents

1 Investigators and Facilities 1
1.1 Study Location: 1
1.2 Study Management: 1
1.3 Principal Investigator 1
1.4 Chief Investigators 1
1.5 Trial Registration 2
2 Background and Rationale 2
2.1 Introduction 2
2.2 Hypothesis in PICO format: 5
3 Study Aims 5
3.1 Primary Aim 5
3.2 Secondary Aims 5
4 Research Plan 6
4.1 Study Design 6
4.1.1 Inclusion Criteria 6
4.1.2 Exclusion Criteria 6
4.1.3 Recruitment and Consent 6
4.1.4 Randomisation 6
4.1.5 Stratification 7
4.1.6 Protocol 7
4.1.7 Umbilical Cord Blood Banking and Delayed Cord Clamping 11
4.1.8 Retrospective Consent 11
4.1.9 Prespecified Conditions for Termination of the Study Protocol 13
4.1.10 Data Collection 13
4.1.11 Feasibility and Timeframe for Study Completion 14
4.2 Sample Size and Data Analysis 14
4.2.1 Primary Outcome 14
4.2.2 Sample Size Calculation 14
4.2.3 Secondary Outcomes 15

4.2.4 Data Analysis 16
5 Ethical Implications 16
6 Study Oversight 17
6.1 Adverse or Unforeseen Events 18
6.2 Interim Analysis 18
7 References 19

1 Investigators and Facilities

1.1 Study Location:

Birth Centre / Operating Theatres of the Royal Women's Hospital (RWH)
Locked Bag 300, Parkville, VIC, 3052

1.2 Study Management:

A multidisciplinary team, consisting of neonatologists, obstetricians, midwives, anaesthesiologists, neonatal nurses, and physiologists will conduct the study.

1.3 Principal Investigators

Dr Shiraz Badurdeen – Neonatal Clinical/Research Fellow
Newborn Research Centre, RWH, shiraz.badurdeen@thewomens.org.au

Dr Douglas BLANK – Neonatal Clinical/Research Fellow
Newborn Research Centre, RWH, douglas.blank@thewomens.org.au

1.4 Chief Investigators

Dr Omar KAMLIN
Neonatal Consultant, RWH, omar.kamlin@thewomens.org.au
Dr Calum ROBERTS
Neonatal Consultant, Monash Children's Hospital, calum.roberts@monash.edu
A/Professor Susan Jacobs
Deputy Clinical Director of Neonatal Services, RWH Sue.jacobs@thewomens.org.au
Dr Marta THIO
Neonatal Consultant, RWH, Marta.ThioLluch@thewomens.org.au
Dr Jennifer DAWSON
Deputy Director of Newborn Research Centre, RWH, Jennifer.Dawson@thewomens.org.au
Dr Stefan KANE
Obstetric Consultant, RWH, Stefan.Kane@thewomens.org.au
A/Prof Alicia DENNIS
Director of Anaesthesiology, RWH, alicia.dennis@thewomens.org.au
A/Prof Susan Donath
Biostatistics, Murdock Children's Institute, Uni Melbourne, susan.donath@mcri.edu.au
A/Prof Graeme POLGLASE
Physiologist, Hudson Institute, Monash University, graeme.polglase@monash.edu
Prof Stuart HOOPER
Physiologist, Director of Ritchie Centre, Monash University, stuart.hooper@monash.edu
Professor Peter DAVIS
Director of Newborn Research Centre, RWH, pgd@unimelb.edu.au

1.5 Trial Registration

Application for registration of the trial will be sought from Australia New Zealand Clinical Trials Registry pending ethics approval and before recruiting the first patient.

2 Background and Rationale

2.1 Introduction

Over 5% percent of all infants born worldwide will need help breathing after birth.[1] In recent studies conducted at the RWH, 7% of term infants received positive pressure ventilation after routine births (RWH Ethics Applications 14/43 and 15/1) and 12% after emergency caesarean deliveries (RWH Ethics Application 16/08).[2 3] Currently, all internationally recognised guidelines state if an infant is not vigorous after birth, the umbilical cord should be clamped and cut so that the infant can be moved to a resuscitation platform where the clinician provides respiratory support via positive pressure ventilation (PPV).[4-7] Resuscitation guidelines are intended for PPV to be effective within 1 minute of birth. Studies show that it takes closer to 2 minutes to achieve effective ventilation.[8 9] During the delay between umbilical cord clamping and effective ventilation, the compromised infant is not receiving oxygen and the heart rate remains dangerously low.

We believe that cutting the umbilical cord to move the compromised newborn infant wastes the opportunity to continue to receive support from the uterine-placental circulation provided by the infant's mother prior to establishing effective ventilation. For months prior to delivery, the placenta provides the developing foetus with oxygen and nutrients. Until lung aeration and pulmonary blood flow is established, the blood that fills the left side of the heart comes preferentially from the umbilical cord. Delaying umbilical cord clamping for just a few minutes after birth while ventilation is being established takes advantage of the placental circulation until the lungs can effectively return blood flow to the left side of the heart and provide oxygen. In addition, for months prior to delivery, the placenta provides the developing foetus with oxygen and nutrients. Birth asphyxia, failure to initiate or sustain spontaneous breathing at birth, claims the lives of more than 800,000 infants every year, the majority of these infants die in resource limited settings. In large interventional studies, implementation of simple resuscitation techniques, like PPV, dropped the rates of death from birth asphyxia by over 40%.[1 10-13] Ventilation prior to umbilical cord clamping may offer the next step in reducing the rates of death from birth asphyxia even further.

Extensive experiments using newborn lambs conducted by our research team demonstrate that establishing effective ventilation prior to umbilical cord clamping improves physiological stability versus cutting the umbilical cord and separating the lambs from the placental circulation prior to establishing ventilation.[14-17] Specifically, lambs receiving ventilation prior to umbilical cord clamping have less bradycardia, less fluctuations in blood pressure, improved systemic oxygenation and cerebral perfusion. However, in humans, improved physiologic stability with delayed cord clamping (DCC) has never been proven It is primarily believed that the major recipient of placental blood is the pulmonary bed. As the infant initiates breathing and establishes lung aeration, the pulmonary blood vessels dilate and the

infant will draw blood from the placenta into the dilated pulmonary blood vessels.[18-21] Infants that received DCC in these studies were breathing spontaneously. If infants do not breathe at birth, guidelines recommend immediately clamping the umbilical cord and moving the infant to a resuscitation platform in order provide positive pressure ventilation (PPV).[6,22,23] Our lab has been unable to substantiate the theory of placental transfusion as the primary mechanism of benefit from DCC, despite extensive controlled experiments. In anaesthetised fetal lambs the onset of mechanical ventilation prior to umbilical cord clamping resulted in a proportional decrease in umbilical venous and arterial flow, but the net flow of blood to the fetal lamb did not change. There was no difference observed in blood volume in the lambs after umbilical cord clamping. In addition, our lab has demonstrated that maternal oxytocin delivery to ewes after delivery, but prior to umbilical cord clamping, restricts umbilical blood flow to newborn lambs, which is consistent with human observations (Stenning et al, presented at PAS 2016 in Baltimore, USA).[24]

Delayed cord clamping without ventilation is recommended for all vigorous infants, but infants that need resuscitation at birth will receive early cord clamping and be moved to a warming bed for positive pressure ventilation.[4 5] The benefits of DCC may be most crucial for the compromised infant. We believe that being able to provide PPV prior to umbilical cord clamping will enable compromised infants to receive the benefits of DCC. In a large, multicentre trial, over 25% of the preterm infants in the DCC group did not receive the intended 60 seconds of DCC because the infants were moved to a warming bed for resuscitation.[25,26]

We believe that compromised infants who have achieved effective ventilation prior to umbilical cord clamping will have improved haemodynamic stability during resuscitation because the placental circulation will continue to provide cardiac preload and gas exchange while pulmonary gas exchange is established. Improved haemodynamic stability in the first minutes of life may decrease the need for intensive resuscitation interventions after birth (e.g. PPV, emergent intubation, and chest compressions) and decrease the risk of significant morbidities, including hypoxic ischaemic encephalopathy and death.

We hypothesise that establishing effective ventilation, either via PPV or effective spontaneous breathing, prior to umbilical cord clamping decreases the incidence of bradycardia in infants born at ≥32 weeks gestational age compared with the current standard of care. We named the intervention: Baby-Directed Umbilical Cord Cutting or Baby-DUCC (RWH ethics 16/34). We have chosen to perform an unblinded, randomised controlled trial to test if Baby-DUCC provides a physiologic advantage. The results of this trial will help us design future RCTs with clinically important outcomes (See Figure).

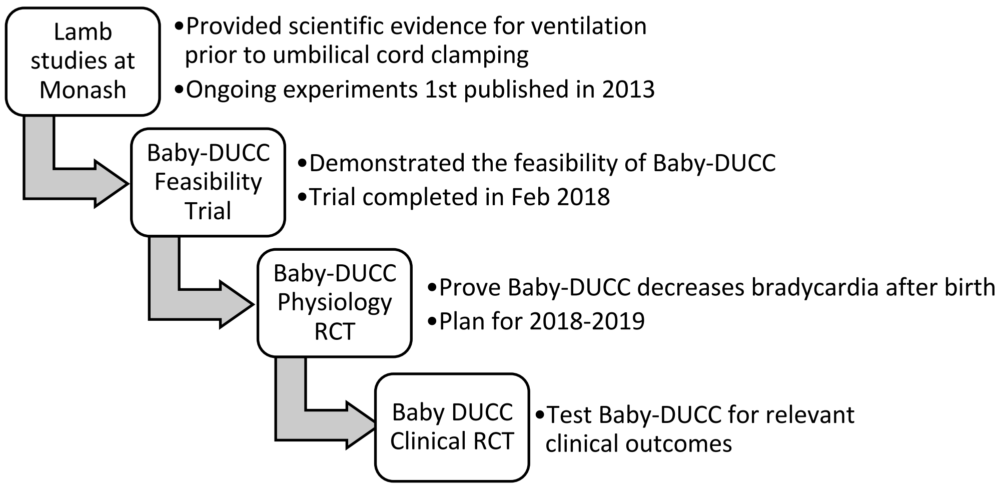

Figure: Strategic plan for testing the Baby-DUCC intervention, development from bench to bedside.

The heart rate and SpO2 of vigorous infants ≥32 weeks receiving delayed cord clamping may be different to established accepted normal values.[27,28] We anticipate that only 10% of the infants in this study will receive resuscitation. We intend to initiate HR and SpO2 monitoring immediately after birth, while the clinical team determining if the infant needs resuscitation. If the infants are vigorous after delivery, they will not be randomised and will receive ≥2 minutes delayed cord clamping. We will collect heart rate and SpO2 values in the first 10 minutes after delivery in vigorous infants who receive ≥2 minutes of delayed cord clamping after birth. We will analyse the observational data of the vigorous infants separately as an observational study.

2.2 Hypothesis in PICO format:

Participants: In infants ≥32 weeks gestational age at birth who require resuscitation at delivery,

Intervention: Does establishing effective ventilation, either via PPV or effective spontaneous breathing, prior to umbilical cord clamping* versus

Comparator: Standard care- immediate cord clamping followed by resuscitation

Outcome: Result in a higher average heart rate between 60-120 seconds after birth.

*In the intervention arm (Baby-DUCC), infants that receive PPV will have umbilical cord clamping ≥1 minute after pedicap/neostat colour change or ≥2 minutes after delivery, which ever time occurs last. Pedicap/neostat colour change indicates exhaled carbon dioxide levels are ≥15mmHg, therefore, the lungs are aerated and pulmonary gas exchange has begun. If the infant is still receiving PPV at 5 minutes, the umbilical cord will be cut and the infant will be moved to the warming bed.

3 Study Aims

3.1 Primary Aim

Our primary aim is to compare the average heart rate between 60-120 seconds after birth in infants who require resuscitation, determined by the attending paediatrician, and receive Baby-Directed Umbilical Cord Cutting versus infants who receive standard of care, immediate cord clamping so that the infant can be moved to the warming bed for resuscitation.

3.2 Secondary Aims

Our secondary aim is to evaluate the differences in other physiologic measures in the delivery room between groups, including incidence of bradycardia in the first 5 minutes after birth, saturation of peripheral oxygen, time to spontaneous breathing and crying, time to pedicap/neostat colour change, time to umbilical cord clamping, need for resuscitation measures and admission to the newborn care unit. We also will investigate short term clinical outcomes for infants and delivering mothers prior to hospital discharge, including comparing rates of potential complications such as maternal bleeding, maternal infection, neonatal jaundice, and neonatal polycythemia. The information gained from the study will serve as the basis to create a randomised controlled trial with measuring clinical outcomes.

Vigorous infants will receive ≥2 minutes of delayed cord clamping, we aim to collect heart rate and SpO2 data in the first 10 minutes after birth in infants that receive delayed cord clamping.

4 Research Plan

4.1 Study Design

This is an unblinded (to the intervention) randomised controlled trial at the Royal Women's Hospital (RWH) and Monash Medical Centre (MMC), Clayton.

4.1.1 Inclusion Criteria

Infants ≥32 weeks' gestation, with a request for a paediatrician to attend the delivery for potential newborn distress, born at the participating centres are eligible for inclusion. The intervention arm (Baby-DUCC) requires that maternal oxytocin administration will occur at between 2 and 5 minutes after delivery. Assessment of the need for early maternal oxytocin administration after delivery, and permission from the maternal care team prior to recruitment and enrolment is required.

4.1.2 Exclusion Criteria

We will not approach expecting mothers of monochorionic twins and multiples of >2, fetuses with known congenital anomalies compromising cardiorespiratory transition after birth, including congenital diaphragmatic hernia, hydrops fetalis, cyanotic congenital heart defects, and airway anomalies that may compromise the ability to provide face mask PPV, and families who are not medicare eligible.

If the maternal treatment team feels that the mother is at high risk for obstetric complications that may be exacerbated by the study intervention, and/or requires early oxytocin administration after delivery, they will not be approached for consent. Potential maternal obstetric complications that may meet criteria for exclusion from the study at the discretion of the maternal care team include abnormal placentation, suspected placental abruption, suspected uterine rupture, significant blood loss, coagulopathy, and previous history of significant blood loss.

4.1.3 Recruitment and Consent

We will obtain verbal consent from the maternal care team to approach expecting mothers for antenatal consent. The researcher will ask the maternal care team to evaluate if there are any maternal risk factors that preclude participation in the study, specifically the risks of postpartum haemorrhage (PPH) if oxytocin is administered between 2 and 5 minutes after birth. Maternal providers include the midwife or obstetrician delivering the infant, and the addition of the anaesthetist in the case of a caesarean delivery. We have successfully used this approach to complete the Baby-DUCC feasibility study (RWH Ethics 16/34, ACTRN12617000610336). In the Baby-DUCC feasibility trial (n=44), 27% of delivering mothers had post-partum haemorrhage (PPH ≥500ml blood loss), median blood loss was 300ml (IQR 300-500), and only 1 mother experienced >1000ml blood loss. The rate of PPH is similar to the historical averages at the RWH. Diamniotic dichorionic twins are eligible for recruitment. Each twin will be randomised independently. Antenatal consent will be sought from mothers expected to deliver at ≥32 weeks.

4.1.4 Randomisation

After delivery, the clinical team will determine if the infant requires resuscitation. During that evaluation, the researcher will initiate HR monitoring. If an infant is determined to need resuscitation, the infant will be randomised to the intervention group (Baby-DUCC) or the control group (immediate cord clamping, and be taken to the warming bed for evaluation). This decision must be made within 60 seconds of delivery of the infant. Computer-generated, random permuted blocks will be used and incorporated into the REDCap randomisation tool (hosted by the Murdoch Children's Research Institute). A weblink to the tool will be accessed via a mobile device at delivery and enables randomisation to be completed within seconds of the decision to commence resuscitation. If the infant is vigorous after birth, the infant will receive ≥2 minutes of delayed cord clamping and we will continue to collect data using ECG, pulse oximetry and/or Doppler ultrasound. Prior to the birth of a study infant the researcher will also review the Baby-DUCC protocol with the maternal and paediatric providers.

4.1.5 Stratification

Infants will be stratified by gestational age (32-35 weeks or ≥36 weeks at birth). Infants ≥36 weeks gestation at birth will also be stratified into non-emergent and emergent deliveries. Emergent deliveries include emergent caesarean sections ("code green") and vaginal instrumental deliveries. We anticipate that 30% of infants recruited will be 32-35 weeks, 35% to be ≥36 weeks delivered emergently, and 35% to be ≥36 weeks delivered non-emergently.

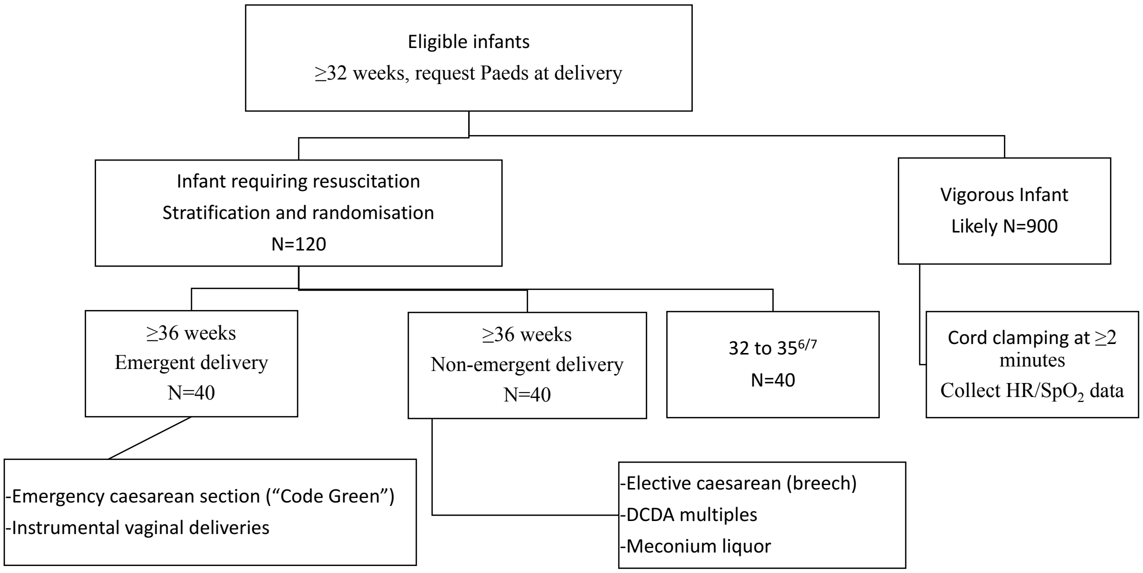

4.1.6 Protocol

All infants will be assessed for the need for respiratory support after birth per Australian Neonatal Resuscitation and RWH guidelines by the clinical team. The researcher will ensure proper stratification and randomisation, obtain vital sign measurements, communicate with the obstetric team regarding

cord clamping and oxytocin administration, and assist the paediatrician in attendance if needed. We do not anticipate that participation in this study will interfere with cuddles after birth, skin-to-skin contact, or breastfeeding after delivery. After a vaginal delivery in both arms of the study, the infant may be evaluated for the need for respiratory support while in the mother's arms.

If the infant requires respiratory support after birth, the infant will be randomised to Baby-DUCC (resuscitation prior to umbilical cord clamping) or the control arm (immediate cord clamping and the infant will be moved to the warming bed for resuscitation measures). If the infant is vigorous and does not need resuscitation, the infant will not be randomised and umbilical cord clamping will occur ≥2 minutes after birth, oxytocin administration will occur after umbilical cord clamping. All monitoring initiated for the purpose of the study will be easily visible to the care team and can be used for evaluation of the infant.

4.1.6.1 Baby-Directed Umbilical Cord Cutting Intervention

The intervention in this study has previously been reviewed and approved by the RWH ethics committee as a feasibility study (Baby-DUCC protocol, RWH Ethics 16/34). If the infant is vigorous and does not need respiratory support, umbilical cord clamping will be delayed until at least 2 minutes after birth. If the non-vigorous infant is randomised to Baby-DUCC, respiratory support will be provided using a portable respiratory support system located on a mobile pole equipped with a T-Piece, oxygen blender, and suction catheter. In a vaginal delivery, the infant will be placed on a portable mattress either on the end of the bed, or if the mother is in stirrups, then the mattress will be placed in a portable cot and positioned between the mother's legs. If respiratory support is indicated, positive pressure ventilation or CPAP, can be provided by mask using the T-Piece attached to the respiratory support pole. A disposal colorimetric exhaled carbon dioxide detector (pedicap or neostat) will be placed between the facemask and T-Piece. Colour change indicates exhaled carbon dioxide ≥15mmHg, and will be monitored by the researcher. If the infant receives PPV, umbilical cord clamping will be delayed until at least 60 seconds after colour change of the pedicap/neostat has occurred. Oxytocin for the prevention of postpartum haemorrhage will be administered after umbilical cord clamping.

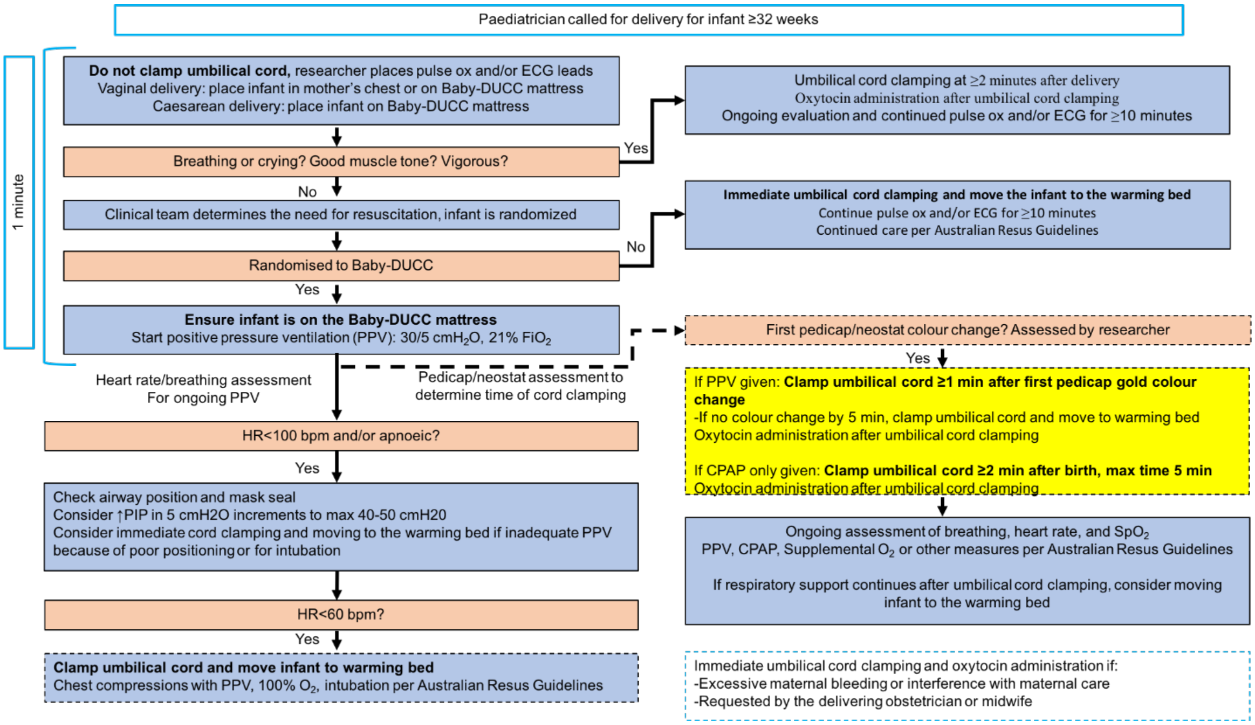

Figure: Protocol for randomisation and resuscitation in the Baby-DUCC RCT

4.1.6.1.1 Sterility at Caesarean Deliveries

In a caesarean section delivery, sterility will be maintained while providing respiratory support and monitoring in the sterile field. The paediatrician and researcher will "scrub in," donning sterile gloves and gowns. Sterilised facemasks, colorimetric CO2 detector, and T-Piece tubing will be used. The infant will be placed on a portable mattress placed on the mother's thighs. To maintain sterility the mattress will be covered with sterile drapes and completely sealed with a sterile Mayo stand cover. Sterile electrocardiograph (ECG) electrodes, pulse oximetry sensor, and/or doppler ultrasound probe covered with a sterile sheath will be used to measure heart rate and/or SpO_2. The cable connecting the sterile electrodes/pulse oximetry sensor will be covered with a sterile ultrasound probe cover. We have successfully developed methods for collecting data and providing respiratory support without disruption of the sterile field after caesarean deliveries maintaining a sterile field in 3 previous studies conducted at the RWH (N=105 infants enrolled, RWH Ethics 14/43, 15/1, and 16/34).[2 3]

Obstetric Staff:
-ON=Circulating RN
-OS=Scrub RN
-O2=OB Reg
-O1=OB Consultant

Neonatal Staff:
-N1=Paediatrician
-R1=Researcher
-N2=RN or midwife
-R2=Researcher

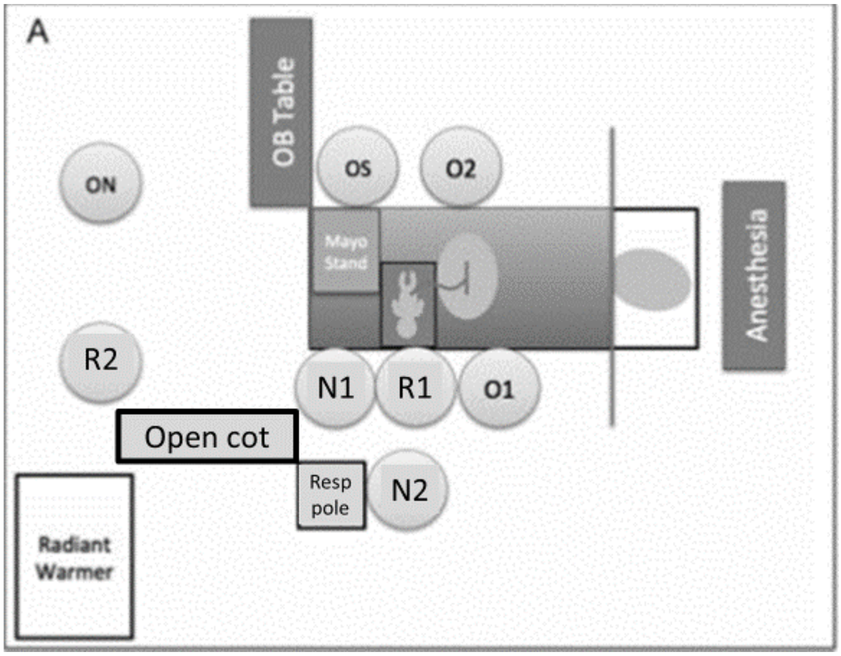

Diagram illustrating where the clinical and research staff will be positioned at a caesarean delivery

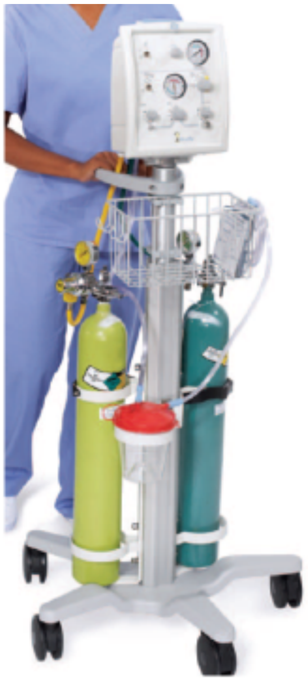

Respiratory support pole with T-Piece, blender, and suction

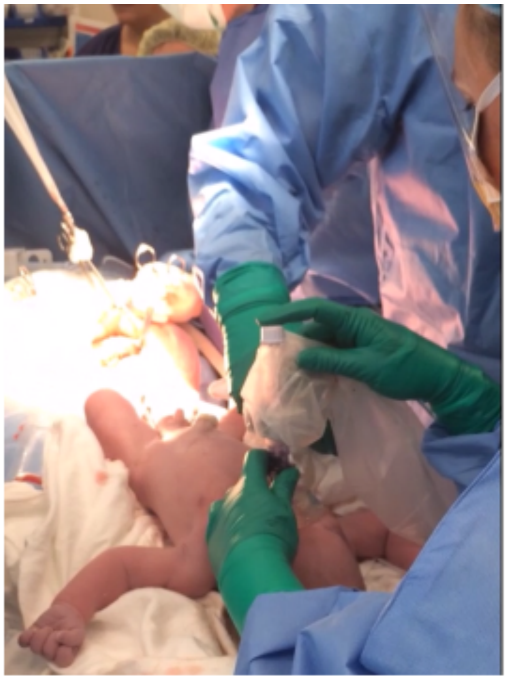

Example of providing PPV prior to umbilical cord clamping after caesarean section from the Baby-DUCC Feasibility Study

4.1.6.2 Control Arm

If the infant is not vigorous and randomised to the control arm, the umbilical cord will be clamped and the infant moved to the warming bed. Resuscitation measures will continue as per the Australian Neonatal Resuscitation guidelines. Oxytocin will be administered after randomisation. Monitoring via ECG, doppler ultrasound and/or pulse oximetry will continue until ≥10 minutes

4.1.7 Umbilical Cord Blood Banking and Delayed Cord Clamping

There are conflicting reports regarding the yield of umbilical cord blood donation after delayed cord clamping.[29,30] In these two studies, there is no comment on the administration of maternal oxytocin after delivery to reduce the risk of post-partum haemorrhage. Our team has demonstrated that oxytocin administration to delivering ewes prior to umbilical cord clamping reduces umbilical blood flow in newborn lambs. Oxytocin administration prior to umbilical cord clamping may be a confounding factor that decreases the yield of umbilical cord blood donation after delayed cord clamping. Therefore, women who wish to participate in the Baby-DUCC study may also consent for umbilical cord blood banking. We plan to compare the results of umbilical cord blood donation as a secondary outcome, if possible.

4.1.8 Retrospective Consent

We will approach expecting mothers for prospective consent in the following scenarios:

- Caesarean sections requiring a paediatrician in attendance with no evidence of fetal distress, including:
    - Elective caesarean section for breech presentation
    - Delivery of dichorionic, diamniotic twins
    - Unplanned caesarean sections no evidence of fetal distress if the expecting mother has an epidural and has adequate pain control
- Vaginal deliveries requiring a paediatrician in attendance with no evidence of fetal distress, including:
    - Meconium stained liquor
    - Delivery of dichorionic, diamniotic twins

We are requesting permission to enrol infants delivered in emergent circumstances with retrospective consent, if prospective consent is not possible. Emergent circumstances that may be appropriate for retrospective consent include:

- Emergency caesarean sections, including “code green” caesarean deliveries and unplanned caesarean sections with evidence of fetal distress
- Instrumental vaginal deliveries

We are concerned that if we require antenatal consent we will not be able to adequately enrol infants delivered emergently, who are at a higher risk for needing resuscitation. Therefore, the results of this study will not be representative of the population of infants born at ≥32 weeks who are most likely to benefit from respiratory support prior to umbilical cord clamping. Prior to emergency caesarean section

or instrumental deliveries there is little opportunity to obtain meaningful informed consent from expecting mothers. Reliance on prospective consent will result in underrepresentation of infants born with distress. If granted retrospective consent, we will review risks with the maternal care team and obtain permission from the maternal care team prior to randomisation. In addition, we will adhere to pre-specified conditions for termination of the study protocol (see below).

4.1.8.1 Precedence for Retrospective Consent at the RWH

Previous studies have demonstrated that studies of high risk populations conducted without retrospective consent underrepresent infants whose mothers have the least education, least antenatal care, and worst delivery outcomes.[31,32] We recently completed the Baby-DUCC Feasibility Trial (RWH Ethics 16/34) in which we have been prepared to provide respiratory support prior to umbilical cord clamping. We have studied 44 infants, obtaining accurate heart rate measurements within 60 seconds of delivery for >90% of enrolled infants. Twenty-three infants delivered via caesarian section, including 6 infants who received resuscitation in the sterile field prior to cord clamping (see photo on the title page). It is our experience that it is feasible to accurately evaluate newborns, obtain accurate physiology data, and provide respiratory support without interfering in the care of the delivering mother or disrupting the sterile field. We approached 10 women for antenatal consent for every 1 neonate that required resuscitation and would have met the inclusion criteria for the Baby-DUCC RCT. The RWH Ethics committee has recently granted retrospective consent for other delivery room trials: SAIL trial, Ethics 14/31 and SEAL trial, Ethics 16/08. The SEAL trial has completed recruitment using retrospective consent of a similar population of infants (≥32 weeks) delivered via emergent caesarean section. In a recently reported UK-based randomised trial of resuscitation with the umbilical cord-intact for very preterm infants, if the delivery was emergent, infants were still randomised and retrospective consent was sought.[33]

4.1.8.2 Balance of Risk

Infants born via emergency caesarean section represent the population of infants born at ≥32 weeks who are most likely to need help breathing after birth. International guidelines for neonatal resuscitation advocate for up to 1 minute of delayed cord clamping if the infant is vigorous.[4 5] Standard clinical guidance at RWH ("Third Stage of Labour – Management" updated 19/01/2016) states that for active management of the third stage of labour, "administer a prophylactic oxytocic agent to the woman with the birth of the anterior shoulder, or within one to two minutes of the birth of the baby" and "early cord clamping: cord clamping which occurs within 2-3 minutes of administration of an oxytocic". While active management is recommended, women who are not at risk of postpartum haemorrhage are offered the choice of physiological management of the third stage, in which the cord is not clamped, and no oxytocin is delivered. The most recent Cochrane Review assessing timing of oxytocin delivery for the third stage of labour[34] concluded that "administration of oxytocin before and after placental expulsion does not significantly influence major clinical outcomes such as the incidence of postpartum haemorrhage", and further noted "Previous evidence (McDonald 2008; Rabe 2004; Soltani 2005) have shown beneficial effects in delaying cord clamping or in cord drainage, and the use of oxytocin after the third stage of labour can add to these benefits".

The Baby-DUCC intervention involves cord clamping and oxytocin administration 2-5 minutes after birth. Women with an increased risk of PPH will be excluded from the study. Furthermore, if any unanticipated risk arises during delivery, we have stipulated that the study protocol be terminated and care provided as per the instructions of the maternal care team (see section 4.1.9 below). We submit that the above evidence indicates that the Baby-DUCC intervention is unlikely to present any difference in risk of post-partum haemorrhage, compared with management strategies of the third stage at the RWH. In the Baby-DUCC feasibility study (N=44), delayed cord clamping and oxytocin administration at 2-5 minutes after birth resulted in a similar incidence of PPH compared to the RWH historical average (results presented at the Maternity Management Meeting on 16th February 2018). In a recently reported UK-based randomised trial in very preterm infants, at least 30% of mothers received a uterotonic agent after cord clamping in the intervention group allocated to ≥2 minutes of delayed cord clamping and resuscitation if required. There was no difference in PPH risk when compared to the control group that received immediate cord clamping with uterotonic administration.[33] Therefore, we feel the Baby-DUCC RCT study fulfils the NHMRC guidelines for approval without prior consent, in individuals highly dependent on medical care where no party is able to give consent. This includes the fact that the project is not controversial, there is a reasonable possibility of benefit over standard care, any risk is justified by the potential benefits to the individual, their inclusion is not contrary to their own interest, and as soon as reasonably possible the infant's family would be informed of their child's inclusion, and would be given the option to withdraw from the study without repercussion.

4.1.9 Prespecified Conditions for Termination of the Study Protocol

If the study is interfering with clinical care of the mother or baby, the intervention will be discontinued (i.e. the umbilical cord will be clamped and the baby will be moved to the warming bed). If the maternal care team feels that prophylactic administration of oxytocin immediately after the delivery of the infant is required, the Baby-DUCC protocol will not be carried out and the umbilical cord will be clamped immediately after delivery. Care for the infant will continue as per the Australian/RWH Neonatal Resuscitation guidelines.

If chest compressions are indicated prior to umbilical cord clamping, the umbilical cord will be clamped immediately. If the treatment team feels that ventilation is inadequate using the portable mattress or intubation is warranted and the baby needs to be moved to the radiant warmer bed to optimize success, the umbilical cord will be clamped and the baby will be moved to the warming bed.

4.1.10 Data Collection

Immediately after birth, a researcher will apply a pulse oximetry sensor and/or ECG leads to obtain heart rate measurements. This may occur prior to randomisation. Doppler ultrasound may be used in conjunction to confirm the heart rate in the event of weak electrical signal or interference. The use of pulse oximetry for infants receiving neonatal resuscitation is routine practice at RWH. We will record the information stored in the monitoring device to a study computer for analysis. A video camera will be focused on the monitor to verify accuracy of the recorded data (plethysmography wave form or QRS complex). Audio recording from the video camera or additional audio recorder will be used to verify

accurately events during delivery, for example the timing of umbilical cord clamping, the time to initiate breathing, and the timing of pedicap/neostat colour change.

The primary outcome will be considered in infants with accurate heart rate values between 60 and 120 seconds after birth. We plan to analyse heart rate every 10 seconds during the study period. Infants included in the primary analysis must have an accurate heart rate measurement in 4/7 data points, including an accurate heart rate by 80 seconds after birth.

Routinely collected clinical data will be used for the secondary outcomes described below.

4.1.11 Feasibility and Timeframe for Study Completion

We anticipate 24 months will be needed to attend the delivery of 1000 infants (42 infants per month) to randomise 120 infants in need of resuscitation. Most infants will be delivered "in-hours," Monday through Friday. Previous studies conducted by our team have provided precedence for the anticipated rates of recruitment:

- Non-Emergent Delivery ≥36 weeks (N=40): 17 attended births/month required, with 10% requiring resuscitation
    - Average recruitment and enrolment rate for both the RISE (RWH Ethics 14/43) and DOLFIN[2,3] (RWH Ethics 15/01) studies was 2-3 infants enrolled per day of recruiting.
    - 7-8% of infants in these studies would have been randomised in the Baby-DUCC RCT
    - 200 working days would be needed to randomise 40 infants with non-emergent delivery.
- Emergent Delivery ≥36 weeks (N=40): 12 attended births/month required, with 16% requiring resuscitation
    - The SEAL trial team (RWH Ethics 16/08) has attended 382 deliveries emergent deliveries in 13 months to enrol 60 infants who needed resuscitation.
    - 12.5% would have been randomised in the Baby-DUCC RCT.
    - The SEAL team averaged 3 days a week of recruitment, all recruitment was "in-hours."
- 32^0 to $35^{6/7}$ (N=40): 12 attended births/month required, with 16% requiring resuscitation
    - In 2017, over 25 eligible infants/month, gestational ages 32^0 to $35^{6/7}$, were born at the RWH.

The heart rates in the first minutes after birth in the referenced trials are unknown. However, 12.5%, 7%, and 7% of the infants in the SEAL, DOLFIN, and RISE trials, respectively received PPV in the delivery room.

4.2 Sample Size and Data Analysis

4.2.1 Primary Outcome

The primary outcome is the average heart rate between 60-120 seconds after birth compared between infants needing resuscitation that are treated using Baby-DUCC and infants that receive immediate cord clamping and transfer to the warming bed. Analysis will be by intention to treat.

4.2.2 Sample Size Calculation

We calculated the sample size to show an improved average heart rate between 60 seconds and 120 seconds in infants that receive resuscitation and are treated with Baby-DUCC compared with infants

that receive immediate cord clamping and resuscitation on a warming bed. The expected HR in the control group is based on an audit of 52 infants born in a 9-month period that received ICC and PPV within 60 seconds of birth and were monitored using a data acquisition system that included ECG electrodes for HR monitoring. Thirty-seven percent of this cohort would have been eligible for the Baby-DUCC RCT. The HR trends for the eligible infants and non-eligible infants do not differ from this cohort. The mean HR from 60-120 seconds after birth was 112 BPM ±29 (SD, standard deviation). In the non-vigorous infants from the Baby-DUCC feasibility trial, the average HR from 60-120 seconds was 149 BPM ±26 SD between 60-120 seconds after birth. We anticipate that the infants in the control arm of the RCT may have higher HR than the infants from the audit because the infants in the audit did not respond to vigorous stimulation and progressed to receiving PPV. Similarly, we anticipate that infants in the Baby-DUCC arm of the RCT may have a lower HR than infants in the feasibility trial because all infants in the RCT will be high risk deliveries (emergency caesarean sections, instrumental births, twins, etc) where many infants in the feasibility trial would be considered low risk births.

Therefore, we hypothesize that infants needing resuscitation who receive Baby-DUCC will have a mean HR of 140 BPM ±30 SD between 60-120 seconds after birth and infants needing resuscitation who receive ICC will have a mean HR of 120 BPM ±30 SD. Accepting a 2-sided alpha<0.05 and 90% power (1-beta), we would need to enrol 49 infants into each arm (N=98). In order to accommodate a 10% attrition rate for detecting the primary outcome due to monitoring failure (based on the Baby-DUCC feasibility pilot study, usually due to poor contact of the ECG leads on the infant), and rounding up to accommodate equal numbers in each stratum, we have increased our total N=120.

4.2.3 Secondary Outcomes

Infant Delivery Room:

-Fetal heart rate prior to delivery if available (via scalp electrode, Doppler, or ultrasound assessment)
-Time to umbilical cord clamping
-Time to 1st cry
- Time to initiate respiratory support (if indicated) from birth
-Time to pedicap/neostat colour change, if the infant receives respiratory support
-Time to regular respirations
-Time to oxytocin delivery to the mother
-Time to obtain accurate data from the ECG or pulse oximeter
-Change in heart rate and oxygen saturation (SpO2) over time between groups
-Proportion of infants with heart rate <100bpm
-Time spent with HR<100bpm
-Time spent with HR>180bpm
-Variability of heart rate for individuals in each group. (Comparison of the standard deviations of heart rate for infants in each group).
-Proportion of infants receiving resuscitation measures: CPAP, PPV, oxygen, intubation, chest compressions
-Maximum fraction of inspired oxygen (FiO_2) and peak inspiratory pressure (PIP) in the delivery room
-Apgar scores at 1, 5, and 10 minutes
-First temperature
-Results of umbilical cord blood gases if available

-Rates of successful umbilical cord blood donation as applicable

Infant Respiratory and General Outcomes, if available:
-Proportion and reason for admitted to NICU/SCN prior to hospital discharge
-Need for respiratory support prior to hospital discharge
-Birth weight
-Feeding at discharge (breast/formula/combination)
-Bilirubin levels, proportion and duration of phototherapy, exchange transfusions
-Haematocrit levels, proportion of infants with polycythaemia and treatment for polycythaemia

Maternal Outcomes:
-Postpartum haemorrhage >500ml, severe haemorrhage >1000ml, need for blood products
-Length of third stage of delivery
-Proportion with retained placenta
-Maternal infections acquired during delivery

4.2.4 Data Analysis

Infant characteristics will be presented as numbers and proportions for categorical variables, means and SDs for normally distributed continuous variables, and medians and IQRs for variables with skewed distribution. Study data will be entered into Excel spreadsheets for management. The information will be merged and statistical analysis will be performed using IBM SPSS, version 24. Pulse oximetry data and ECG will only be used if the signal is determined to be accurate based on visible video recording of plethysmograph wave form or QRS complex that shows good quality signal, or concurrent Doppler ultrasound heartbeat.

For the primary outcome, the average heart rate between 60-120 seconds after birth for each patient will be calculated, and compared between intervention groups using an independent t-test. Imbalances in the randomised strata will be adjusted for using linear regression. Continuous heart rate and SpO_2 values upto the first 10 minutes after delivery will be collected, and trends compared between groups using two-way mixed ANOVA or linear mixed methods regression depending on completeness of data. Continuous variables will be compared using an independent samples t-test if normally distributed and a 2-sided Mann-Whitney U test for non-normally distributed data. Categorical variables will be compared using the Fisher's exact test. Statistical significance will be considered at $p<0.05$. Subgroup analyses for each stratum are planned for the primary outcome.

5 Ethical Implications

For non-emergent deliveries, after receiving permission from the maternal care team to recruit expecting mothers, we will seek written antenatal consent for participation in this study. For emergency deliveries, we will review risks with the maternal care team and obtain permission from the maternal care team prior to the delivery. We will seek permission from the delivering mother for inclusion in the study at the first appropriate time.

Data collected in this study will be de-identified and stored securely. Participation in the study is voluntary and reimbursement will not be provided. Women are free to revoke consent at any time and if they do so, they will be assured that declining to continue to participate in the study will not interfere in their care or in the care of their infant.

6 Study Oversight

An independent Data Monitoring and Safety Committee (DMSC) will be established for the Baby-DUCC Physiology RCT, consisting of at least two independent clinicians (neonatologist, midwife, or obstetrician) and one independent statistician. This committee will perform an interim safety analysis, monitoring of adverse events, compliance with trial protocol, and progress of recruitment.

6.1 Adverse or Unforeseen Events

We plan to have our data reviewed by the DMSC for adverse maternal and neonatal outcomes after the primary outcome is available for 30% (N=30) and 50% (N=51) of the study sample. Specifically, we will be comparing the incidence of potentially avoidable adverse event between study arms, including:

Maternal adverse events:

- Maternal PPH and the need for transfusions
- Maternal infection

Neonatal adverse events:

- Proportion of infants who are intubated
- Proportion of infants who receive chest compressions
- Admissions to the NICU
- Pneumothorax
- Jaundice treated with either phototherapy or exchange transfusion
- Polycythaemia requiring treatment

6.2 Interim Analysis

We plan to have our data reviewed for adverse maternal and neonatal outcomes after the primary outcome is known for 51% (N=52) of the infants by the DMSC. Recommendations for change in the sample size may be recommended if a substantial disparity is noted. The relative risk for major outcomes will be examined in the two groups.

7 References

1. Lee AC, Cousens S, Wall SN, et al. Neonatal resuscitation and immediate newborn assessment and stimulation for the prevention of neonatal deaths: a systematic review, meta-analysis and Delphi estimation of mortality effect. BMC public health 2011;11 Suppl 3:S12. doi: 10.1186/1471-2458-11-S3-S12

2. Blank DA, Rogerson SR, Kamlin COF, et al. Lung ultrasound during the initiation of breathing in healthy term and late preterm infants immediately after birth, a prospective, observational study. Resuscitation 2017;114:59-65. doi: 10.1016/j.resuscitation.2017.02.017

3. Blank DA, Kamlin COF, Rogerson SR, et al. Lung ultrasound immediately after birth to describe normal neonatal transition: an observational study. Archives of disease in childhood Fetal and neonatal edition 2017 doi: 10.1136/archdischild-2017-312818

4. Perlman JM, Wyllie J, Kattwinkel J, et al. Part 7: Neonatal Resuscitation: 2015 International Consensus on Cardiopulmonary Resuscitation and Emergency Cardiovascular Care Science With Treatment Recommendations. Circulation 2015;132(16 Suppl 1):S204-41. doi: 10.1161/CIR.0000000000000276

5. Wyckoff MH, Aziz K, Escobedo MB, et al. Part 13: Neonatal Resuscitation: 2015 American Heart Association Guidelines Update for Cardiopulmonary Resuscitation and Emergency Cardiovascular Care. Circulation 2015;132(18 Suppl 2):S543-60. doi: 10.1161/CIR.0000000000000267

6. Singhal N, Lockyer J, Fidler H, et al. Helping Babies Breathe: global neonatal resuscitation program development and formative educational evaluation. Resuscitation 2012;83(1):90-6. doi: 10.1016/j.resuscitation.2011.07.010

7. Wyllie J, Bruinenberg J, Roehr CC, et al. European Resuscitation Council Guidelines for Resuscitation 2015: Section 7. Resuscitation and support of transition of babies at birth. Resuscitation 2015;95:249-63. doi: 10.1016/j.resuscitation.2015.07.029

8. Hooper SB, Fouras A, Siew ML, et al. Expired CO2 levels indicate degree of lung aeration at birth. PloS one 2013;8(8):e70895. doi: 10.1371/journal.pone.0070895

9. Blank D, Rich W, Leone T, et al. Pedi-cap color change precedes a significant increase in heart rate during neonatal resuscitation. Resuscitation 2014;85(11):1568-72. doi: 10.1016/j.resuscitation.2014.08.027

10. Little GA, Keenan WJ, Niermeyer S, et al. Neonatal Nursing and Helping Babies Breathe: An Effective Intervention to Decrease Global Neonatal Mortality. Newborn and Infant Nursing Reviews 2011;11(2):82-87. doi: 10.1053/j.nainr.2011.04.007

11. Msemo G, Massawe A, Mmbando D, et al. Newborn mortality and fresh stillbirth rates in Tanzania after helping babies breathe training. Pediatrics 2013;131(2):e353-60. doi: 10.1542/peds.2012-1795

12. Carlo WA, Goudar SS, Pasha O, et al. Neurodevelopmental outcomes in infants requiring resuscitation in developing countries. The Journal of pediatrics 2012;160(5):781-5 e1. doi: 10.1016/j.jpeds.2011.10.007

13. Goudar SS, Somannavar MS, Clark R, et al. Stillbirth and newborn mortality in India after helping babies breathe training. Pediatrics 2013;131(2):e344-52. doi: 10.1542/peds.2012-2112

14. Bhatt S, Alison BJ, Wallace EM, et al. Delaying cord clamping until ventilation onset improves cardiovascular function at birth in preterm lambs. The Journal of physiology 2013;591(Pt 8):2113-26. doi: 10.1113/jphysiol.2012.250084

15. Polglase GR, Dawson JA, Kluckow M, et al. Ventilation onset prior to umbilical cord clamping (physiological-based cord clamping) improves systemic and cerebral oxygenation in preterm lambs. PloS one 2015;10(2):e0117504. doi: 10.1371/journal.pone.0117504

16. Blank DA, Polglase GR, Kluckow M, et al. Haemodynamic effects of umbilical cord milking in premature sheep during the neonatal transition. Archives of disease in childhood Fetal and neonatal edition 2017 doi: 10.1136/archdischild-2017-314005

17. Polglase GR, Blank DA, Barton SK, et al. Physiologically based cord clamping stabilises cardiac output and reduces cerebrovascular injury in asphyxiated near-term lambs. Archives of disease in childhood Fetal and neonatal edition 2017 doi: 10.1136/archdischild-2017-313657

18. Rabe H. Effect of timing of umbilical cord clamping and other strategies to influence placental transfusion at preterm birth on maternal and infant outcomes. Cochrane Database Syst Rev. 2012(8) doi: 10.1002/14651858

19. Ghavam S, Batra D, Mercer J, et al. Effects of placental transfusion in extremely low birthweight infants: meta-analysis of long- and short-term outcomes. Transfusion 2014;54(4):1192-98. doi: 10.1111/trf.12469

20. Kaempf JW, Tomlinson MW, Kaempf AJ, et al. Delayed umbilical cord clamping in premature neonates. Obstetrics and gynecology 2012;120(2 Pt 1):325-30. doi: 10.1097/AOG.0b013e31825f269f

21. Mercer JS, Erickson-Owens DA, Vohr BR, et al. Effects of Placental Transfusion on Neonatal and 18 Month Outcomes in Preterm Infants: A Randomized Controlled Trial. The Journal of pediatrics 2016;168:50-5 e1. doi: 10.1016/j.jpeds.2015.09.068

22. Kattwinkel J, Perlman J. The Neonatal Resuscitation Program: The Evidence Evaluation Process and Anticipating Edition 6. NeoReviews 2010;11(12):e673-e80. doi: 10.1542/neo.11-12-e673

23. Perlman JM, Wyllie J, Kattwinkel J, et al. Neonatal resuscitation: 2010 International Consensus on Cardiopulmonary Resuscitation and Emergency Cardiovascular Care Science with Treatment Recommendations. Pediatrics 2010;126(5):e1319-44. doi: 10.1542/peds.2010-2972B

24. Boere I, Roest AA, Wallace E, et al. Umbilical blood flow patterns directly after birth before delayed cord clamping. Archives of disease in childhood Fetal and neonatal edition 2015;100(2):F121-5. doi: 10.1136/archdischild-2014-307144

25. Popat H, Robledo KP, Sebastian L, et al. Effect of Delayed Cord Clamping on Systemic Blood Flow: A Randomized Controlled Trial. The Journal of pediatrics 2016;178:81-86 e2. doi: 10.1016/j.jpeds.2016.08.004

26. Tarnow-Mordi W, Morris J, Kirby A, et al. Delayed versus Immediate Cord Clamping in Preterm Infants. The New England journal of medicine 2017;377(25):2445-55. doi: 10.1056/NEJMoa1711281

27. Dawson JA, Kamlin CO, Wong C, et al. Changes in heart rate in the first minutes after birth. Archives of disease in childhood Fetal and neonatal edition 2010;95(3):F177-81. doi: 10.1136/adc.2009.169102

28. Dawson JA, Kamlin CO, Vento M, et al. Defining the reference range for oxygen saturation for infants after birth. Pediatrics 2010;125(6):e1340-7. doi: 10.1542/peds.2009-1510

29. Allan DS, Scrivens N, Lawless T, et al. Delayed clamping of the umbilical cord after delivery and implications for public cord blood banking. Transfusion 2016;56(3):662-5. doi: 10.1111/trf.13424

30. Frandberg S, Waldner B, Konar J, et al. High quality cord blood banking is feasible with delayed clamping practices. The eight-year experience and current status of the national Swedish Cord Blood Bank. Cell Tissue Bank 2016;17(3):439-48. doi: 10.1007/s10561-016-9565-6

31. Rich WD, Auten KJ, Gantz MG, et al. Antenatal consent in the SUPPORT trial: challenges, costs, and representative enrollment. Pediatrics 2010;126(1):e215-21. doi: 10.1542/peds.2009-3353

32. Rich W, Finer NN, Gantz MG, et al. Enrollment of extremely low birth weight infants in a clinical research study may not be representative. Pediatrics 2012;129(3):480-4. doi: 10.1542/peds.2011-2121

33. Duley L, Dorling J, Pushpa-Rajah A, et al. Randomised trial of cord clamping and initial stabilisation at very preterm birth. Archives of Disease in Childhood Fetal and Neonatal Edition 2018;103:F6–F14. doi:10.1136/archdischild-2016-312567

34. Soltani H, Hutchon DR, Poulose TA. Timing of prophylactic uterotonics for the third stage of labour after vaginal birth. Cochrane Database Syst Rev 2010(8):CD006173. doi: 10.1002/14651858.CD006173.pub2
