# Supplementary material for: Physiologically based cord clamping for infants ≥32+0 weeks gestation: A randomised clinical trial and reference percentiles for heart rate and oxygen saturation for infants ≥35+0 weeks gestation
Source: PLoS Med. 2022 Jun 23;19(6):e1004029. doi: 10.1371/journal.pmed.1004029 (PMC9269938; doi:10.1371/journal.pmed.1004029)
Supplement: S4 Supporting information — Table A. Summary statistics and selected group differences (where estimable) for safety outcomes. CI, confidence interval; ECC, early cord clamping; L, litre; PBCC, physiologically based cord clamping. Table B. Percentiles tables for heart rate and oxygen saturation for all births. Table C. Percentiles tables for heart rate and oxygen saturation for cesarean births. Table D. Percentiles tables for heart rate and oxygen saturation for vaginal births. Fig A. (A) Individual heart rate trajectories for infants with heart rate >200 beats per minute (bpm) within 2 minutes of birth and (B) infants with heart rate <100 bpm within 2 minutes of birth. Fig B. (A) Individual trajectories of oxygen saturation (SpO2) for infants with SpO2 >90% within 2 minutes of birth and (B) infants with SpO2 <50% within 2 minutes of birth. (PDF) [file pmed.1004029.s004.pdf]

Supplementary Material

Supplement to: Badurdeen S, Davis PG, Hooper SB et al. Physiologically-based cord clamping for infants ≥ 32^{+0} weeks gestation - a randomised clinical trial and reference percentiles for heart rate and oxygen saturation.

Contents

	Pages
1. Supplemental Table A. Safety Outcomes	2
2. Percentiles tables for heart rate and oxygen saturation	3-4
3. Supplemental Figures	5-6

Supplemental Table A. Summary statistics and selected group differences (where estimable) for safety outcomes.

Supplemental Table A: Safety outcomes

	PBCC arm (N = 63)	ECC arm (N = 60)	Risk Difference (95% CI)
**Maternal**			
Post-partum haemorrhage ≥1 L or the need for blood transfusion	6 (10%)	5 (8%)	1.2% (-8.9%, 11.3%)
Maternal post-partum haemorrhage between 500ml-999ml without blood transfusion	15 (24%)	14 (23%)	0.5% (-14.5%, 15.5%)
Critical care admission	0 (0%)	1 (2%)	
Infection up to 30 days after caesarean birth only^	1 (4%) N=27	0 (0%) N=25	
Thromboembolic event up to 30 days after caesarean birth	0 (0%) N=27	0 (0%) N=25	
Maternal death	0 (0%)	0 (0%)	
**Infant**			
Intubation in the delivery room	0 (0%)	0 (0%)	
Chest compressions in the delivery room	0 (0%)	0 (0%)	
Admissions to Neonatal Intensive Care for infants ≥36 weeks' gestation*	2 (4%) N=56	1 (2%) N=54	
First temperature <36.5 degrees Celsius	13 (21%)	10 (17%)	4.0% (-9.8%, 17.7%)
First temperature <35.5 degrees Celsius	1 (2%)	1 (2%)	
Pneumothorax treated with needle aspiration and/or chest tube insertion	0 (0%)	0 (0%)	
Jaundice treated with exchange transfusion	0 (0%)	0 (0%)	
Polycythaemia requiring partial exchange transfusion	0 (0%)	0 (0%)	
Hypoxic ischaemic encephalopathy treated with therapeutic hypothermia	0 (0%)	1 (2%)	
Significant acquired brain injury (on imaging or clinical diagnosis) as documented by the clinical team	0 (0%)	0 (0%)	
Neonatal death	0 (0%)	0 (0%)	

^ Maternal post-caesarean infection was defined as maternal hospital re-attendance and commencement of antibiotic therapy (oral or intravenous) by emergency or maternity clinical teams.

*Does not include admissions to special care nursery.

Note, no adjustment for the randomisation stratification factors is made in the risk difference.

Abbreviations: CI, confidence interval; ECC, early cord clamping; L, litre; PBCC, physiologically-based cord clamping.

Percentiles tables for heart rate and oxygen saturation (SpO_2) for infants ≥35^{+0} weeks' gestation and no respiratory support after birth.

Supplemental Table B. Percentiles tables for heart rate and oxygen saturation for all births.

All births N=295	Centile	97	90	75	50	25	10	3
	Minutes							
Heart Rate (bpm)	1	213	200	186	171	156	143	130
	2	212	200	187	173	158	146	133
	3	210	198	186	172	158	146	134
	4	208	196	184	171	157	145	133
	5	205	194	182	169	156	144	132
	6	203	192	180	167	153	141	130
	7	201	190	177	164	151	139	127
	8	200	188	175	162	148	135	123
	9	199	186	173	159	144	132	119
	10	198	185	171	156	141	127	114
SpO_2 (%)	1	92	84	75	66	56	47	39
	2	98	90	82	73	63	55	47
	3	100	96	89	81	73	66	58
	4	100	99	93	86	79	72	66
	5	100	100	95	89	83	78	72
	6	100	100	97	91	86	82	77
	7	100	100	98	93	89	85	81
	8	100	100	99	95	91	87	83
	9	100	100	100	96	92	88	85
	10	100	100	100	97	93	89	85

Supplemental Table C. Percentiles tables for heart rate and oxygen saturation for caesarean births.

Caesarean Births N=134	Centile	97	90	75	50	25	10	3
	Minutes							
Heart Rate (bpm)	1	206	193	180	165	150	137	124
	2	206	194	181	167	153	141	129
	3	204	192	180	167	153	141	130
	4	201	190	178	165	152	141	129
	5	199	187	176	163	150	139	128
	6	196	185	173	161	148	137	125
	7	194	183	171	158	145	133	122
	8	192	180	168	155	142	130	118
	9	190	178	166	152	138	126	114
	10	189	176	163	149	134	121	108
SpO_2 (%)	1	77	68	59	50	40	31	22
	2	94	87	79	70	61	53	45
	3	100	93	86	79	71	64	57
	4	100	97	91	84	77	71	65

	5	100	99	94	88	82	76	71
	6	100	100	95	90	85	81	76
	7	100	100	97	92	88	84	80
	8	100	100	98	94	90	86	83
	9	100	100	100	95	91	88	84
	10	100	100	100	97	92	88	84

Supplemental Table D. Percentiles tables for heart rate and oxygen saturation for vaginal births.

Vaginal Births N=161	Centile	97	90	75	50	25	10	3
	Minutes							
Heart Rate (bpm)	1	217	204	191	176	161	148	135
	2	216	203	191	177	163	151	138
	3	213	202	190	176	163	151	139
	4	211	199	188	175	162	150	139
	5	208	197	186	173	160	149	138
	6	206	195	184	171	158	147	136
	7	205	193	182	169	156	144	133
	8	203	192	180	167	153	141	130
	9	203	190	178	164	150	138	126
	10	202	189	176	162	147	134	121
SpO_2 (%)	1	84	76	67	57	47	38	29
	2	99	91	83	74	66	58	50
	3	100	97	90	82	74	67	60
	4	100	100	94	87	80	74	68
	5	100	100	96	90	84	79	74
	6	100	100	97	92	87	83	78
	7	100	100	99	94	90	86	82
	8	100	100	100	96	91	88	84
	9	100	100	100	97	93	89	85
	10	100	100	100	98	93	90	86

Supplemental Figures

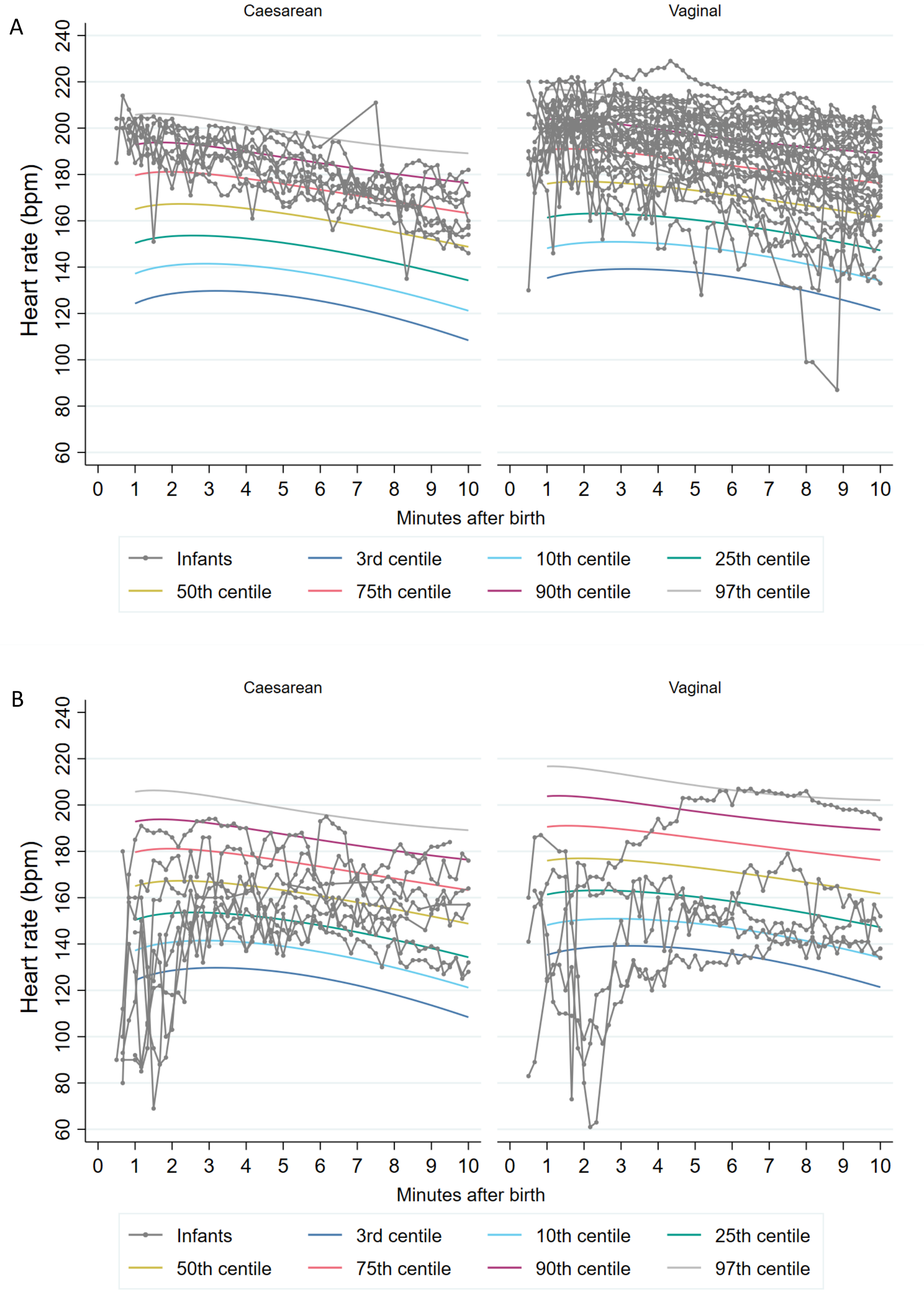

Supplemental Fig A. (A) Individual heart rate trajectories for infants with heart rate >200 beats per minute (bpm) within 2 minutes of birth and (B) infants with heart rate <100 bpm within 2 minutes of birth.

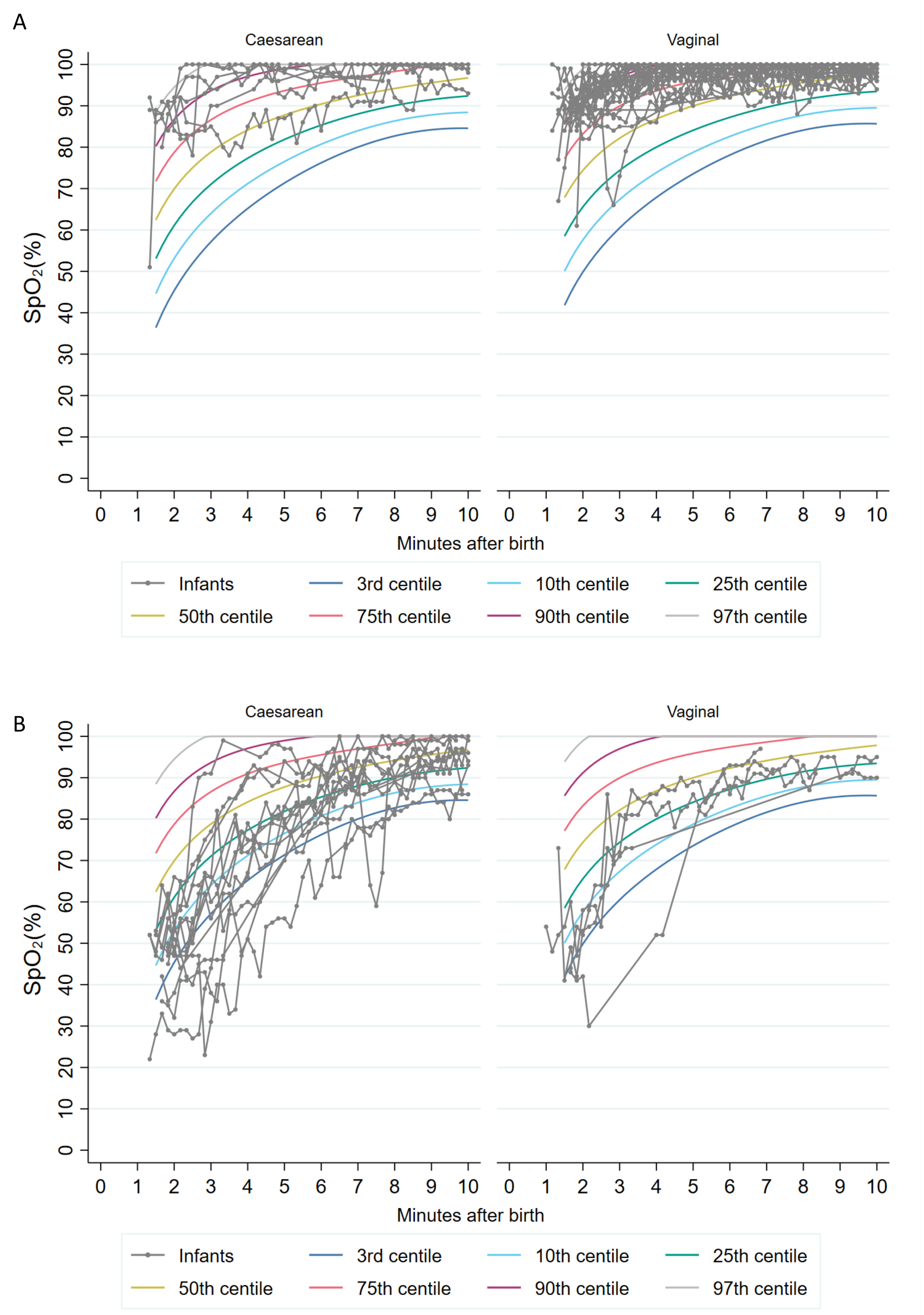

Supplemental Fig B. (A) Individual trajectories of oxygen saturation (SpO_2) for infants with $SpO_2 > 90\%$ within 2 minutes of birth and (B) infants with $SpO_2 < 50\%$ within 2 minutes of birth.
