# Supplementary material for: Physiologically based cord clamping for infants ≥32+0 weeks gestation: A randomised clinical trial and reference percentiles for heart rate and oxygen saturation for infants ≥35+0 weeks gestation
Source: PLoS Med. 2022 Jun 23;19(6):e1004029. doi: 10.1371/journal.pmed.1004029 (PMC9269938; doi:10.1371/journal.pmed.1004029)
Supplement: Attachment — Submitted filename: BDRCT_Response to reviewers.docx [file pmed.1004029.s005.docx]

**Response to reviewers**

*On behalf of the co-authors of this PBCC trial, we sincerely thank you for your thorough review and astute comments, questions, and suggestions. The result will be a strengthened manuscript that we hope will be suitable for publication. We have incorporated all suggestions in track changes in the manuscript or have addressed the suggestion/questions with a response in italics below. Please let us know if you have any further concerns.*

*Kind regards,*

*Shiraz Badurdeen, on behalf of the co-authors.*

**Requests from the editors:**

General comments:

-Please include line numbers in your revised manuscript, ideally not starting from 1 with each new page.

*Line numbers have now been included.*

-Throughout the paper, please adapt reference call-outs to the following style: "... newborn mortality worldwide [1,2]." (noting the absence of spaces within the square brackets).

*This has been adapted as requested.*

-Data availability:

Please provide a web address for accessing the Monash University Research Repository.

*Data is published in the Monash University Research Repository at the following web address. It is currently under embargo and will be released upon acceptance of the manuscript.*

*https://doi.org/10.26180/19579603.v1*

-Title: Please revise your title according to PLOS Medicine's style. Please place the study design in the subtitle (ie, after a colon). We suggest “Physiologically-based cord clamping for infants ≥ 32+0 weeks gestation: A randomised clinical trial” or similar

*This colon has been incorporated into the revised manuscript. We feel that the inclusion of the reference percentiles in the title signposts important data from the non-randomised infants and will attract readers particularly interested in the percentiles.*

*“Physiologically-based cord clamping for infants ≥ 32+0 weeks gestation: a randomised clinical trial and reference percentiles for heart rate and oxygen saturation.”*

Abstract:

-Please report your abstract according to CONSORT for abstracts, following the PLOS

Medicine abstract structure (Background, Methods and Findings, Conclusions): <http://www.consort-statement.org/extensions?ContentWidgetId=562> Please combine the Methods and Findings sections into one section, “Methods and Findings”.

*This suggestion has been incorporated into the revised manuscript.*

-Abstract Background: Please expand upon the context of why the study is important.

*This suggestion has been incorporated into the revised manuscript.*

*“Globally, the majority of newborns requiring resuscitation at birth are full term or late-preterm infants. These infants typically have their umbilical cord clamped early (ECC) to move to a resuscitation platform, losing the potential support of the placental circulation. Physiologically-based cord clamping (PBCC) is clamping the umbilical cord after establishing lung aeration and holds promise as a readily available means of improving early newborn outcomes. In mechanically ventilated lambs, PBCC improved cardiovascular stability and reduced hypoxia."*

Abstract Methods and Findings:

-Please provide brief demographic details of the study population (e.g. sex, gestational age, etc)

*This suggestion has been incorporated and we have added a statement in the abstract.*

*“Median [interquartile range, IQR] for gestational age was 39.9 [38.3-40.7] weeks in PBCC infants and 39.6 [38.4-40.4] weeks in ECC infants. Forty-nine percent and 50% of the PBCC and ECC infants were female, respectively.”*

-Please specify to which study arms the five infants with missing primary outcome data belonged to.

*This suggestion has been incorporated into the revised abstract.*

*“Five infants (PBCC=2, ECC=3, 4% total) had missing primary outcome data.”*

-Please define IQR and CI at first use

*This has been incorporated into the revised abstract.*

-Please be more specific as what ‘normative data’ is in the last sentence of the Abstract Methods and Findings section

*We have added the requested statements to the abstract.*

*“Data from these infants was used to create percentile charts of expected HR and oxygen saturation in vigorous infants receiving DCC.”*

-Please describe 2-3 of the main limitations of the study's methodology."

*We have added the requested statements to the abstract.*

*“The study was limited by the small number of infants requiring prolonged or advanced resuscitation. PBCC may provide other important benefits we did not measure, including improved maternal-infant bonding and higher iron stores.”*

Abstract Conclusions:

-Please begin your Abstract Conclusions with "In this study, we observed ..." or similar, and summarize the main findings from your study, without overstating your conclusions. Please emphasize what is new and address the implications of your study, being careful to avoid assertions of primacy.

*We have added the requested statements to the abstract:*

*“In this study, we observed that PBCC resulted in similar mean HR compared to infants receiving ECC. The findings suggest that for infants ≥32+0 weeks’ gestation who receive brief, effective resuscitation at closely monitored births, PBCC has limited benefits over ECC in terms of key physiological markers of transition. PBCC was feasible using a simple, low-cost strategy at both caesarean and vaginal births. The percentile charts of HR and oxygen saturation may guide clinicians monitoring the transition of at-risk infants who receive DCC.”*

Author Summary:

-Under ‘Why was this study done?’, please add a final bullet point outlining your study question.

*We have added the requested statements to the author summary section.*

*“- We aimed to determine whether PBCC provides physiological benefits over ECC for infants requiring resuscitation at birth. We also aimed to update the reference percentiles of heart rate and oxygen saturation in vigorous infants receiving DCC.”*

-In the final bullet point of ‘What Do These Findings Mean?’, please describe the main limitations of the study in non-technical language.

*We have added the requested statements to the author summary section.*

*“-Limitations include the small number of infants requiring prolonged or advanced resuscitation, reflecting the presence of close fetal monitoring and skilled newborn resuscitation providers. PBCC may provide other important benefits that we did not measure, including improved maternal-infant bonding and higher iron stores.”*

Methods:

-Please add the following statement, or similar, to the Methods: "This study is reported as per the Consolidated Standards of Reporting Trials (CONSORT) guideline (S1 Checklist)." When completing the checklist, please use section and paragraph numbers, rather than page numbers which will likely no longer correspond to the appropriate sections after copy-editing.

*This has now been edited as suggested*

-Please refer to the supplementary file name for your statistical analysis plan.

*This has now been added.*

Results:

-Under ‘Secondary outcomes’ paragraph, please be clear whether the results presented are mean or median e.g. time to initiation of respiratory support

*Corrected in the results section.*

Discussion:

-Please present and organize the Discussion as follows:

1. A short, clear summary of the article's findings

2. What the study adds to existing research

3. Where and why the results may differ from previous research

4. Strengths and limitations of the study

5. Implications and next steps for research, clinical practice, and/or public policy

6. one-paragraph conclusion.

*The Discussion has now been re-organised as suggested.*

-Please remove all subheadings within your Discussion e.g. Limitations and other considerations

*There are no subheadings in the Discussion section of the revised manuscript.*

Figures:

-Please define all abbreviations used in the figure legend, including in the Supporting Information files.

*All abbreviations have now been defined.*

-Please consider avoiding the use of red and green together in order to make your figure more accessible to those with colour blindness.

*The percentile charts have now been changed to be more accessible to those with colour blindness.*

Tables:

-When a p value is given, please specify the statistical test used to determine it in the table legend.

*The relevant statistical test is now provided in the table legend. We also noticed a typo in the p-value for the primary outcome. This has now been changed throughout the manuscript from 0.29 to 0.39.*

***^“‡^*** *Calculated from a linear regression model adjusted for the randomisation stratification factors.”*

-Table 3 has been mislabelled as Table 2

*We cannot find the mis-labelled table.*

Please define abbreviations used in the table legend of each table, including in the Supporting Information files.

*All abbreviations used have now been defined.*

References:

-Please ensure that journal name abbreviations match those found in the National Center for Biotechnology Information (NCBI) databases (http://www.ncbi.nlm.nih.gov/nlmcatalog/journals), and are appropriately formatted and capitalised.

*The references have been formatted as requested.*

-Please name 6 authors prior to ‘et al’, and please do not use italics for journal names.

Please also see https://journals.plos.org/plosmedicine/s/submission-guidelines#loc-references for further details on reference formatting.

*The references have been formatted as requested.*

**Comments from the reviewers:**

**Reviewer #1: Statistical review**

This paper reports a randomised controlled trial comparing two approaches to resuscitation of newborn infants, it also reports updated reference charts for heart rate and oxygen saturation in newborns.

Generally the trial was well reported. I have some minor comments.

1. Abstract: postpartum hemorrhage is one of many secondary outcomes, so it is not clear why it is highlighted in the abstract. Guidance typically is to not selectively report secondary outcomes, so I would either only include primary and safety outcome, or add more about how many secondary outcomes there were (31 on the registration page) and summary of which showed differences (e.g. 'x of the 31 secondary outcomes were statistically significant...').

*Thank you for your comment. As per the CONSORT extension for Abstracts, we intended to include post-partum hemorrhage (PPH) as the most important adverse event/side effect. PPH was closely monitored by the DSMB and is the major safety concern for clinicians at high-risk births when cord clamping and uterotonic administration are deferred. As the reviewer suggests, we have now included the total number of secondary safety outcomes in the abstract. We did not perform statistical tests of significance between groups for the secondary outcomes, as specified in the Statistical Analysis Plan.*

*“Among 31 secondary outcomes, post-partum haemorrhage ≥500 ml occurred in 34% and 32% of mothers in the PBCC and ECC arms respectively.”*

2. Methods: I would recommend that it is made clear what the primary and secondary outcomes of the study were, perhaps in an 'Outcomes' subsection. If ones that were included in the registration and protocol are not reported here then please briefly explain why, or that they will be reported separately.

*Thank you. An Outcomes subsection is now included in the Methods.*

*“****Outcomes***

*The primary outcome was mean HR between 60-120 seconds after birth determined by 3-lead ECG. For each infant, the mean of the HR measurements at 10 second intervals between 60-120 seconds after birth was calculated, providing HR data was available at four data points including at least one data point by 80 seconds after birth. We pre-specified 31 secondary outcomes as detailed in the Statistical Analysis Plan. An additional secondary outcome was specified in the trial registration, “Rates of successful umbilical cord blood donation as applicable”. However, cord blood donation did not occur for infants recruited in the trial. Therefore, we have no data to report on this outcome.”*

3. Statistical analysis: where 'strata' is mentioned, I would clarify this is 'randomisation strata'.

*Thanks, this has been corrected.*

4. Statistical analysis: When binary regression is mentioned, I would clarify which type (I presume not logistic regression as risk differences are reported).

*Binomial regression was used and has now been specified.*

5. Statistical analysis: what outcomes (or types of outcomes) was quantile regression used for? This is explained in the SAP but I would briefly summarise it in the paper.

*Thank you. This has now been specified more clearly.*

*“For secondary outcomes, comparison between the treatment arms was estimated using binomial regression (dichotomous outcomes), linear regression (where the summary measure is the mean) or quantile regression (where the summary measure is the median), adjusted for the stratification factors used during randomisation.”*

6. Results, page 13: one of the binary outcomes (receiving of PPV) doesn't include the estimated risk difference and CIs in the text.

*As specified in the Statistical Analysis Plan (Table 3, page 21), we did not calculate a risk difference and CI’s for this outcome. PPV was considered a subset of the secondary outcome “Any resuscitation in the delivery room” for which the risk difference and CI’s were calculated and reported.*

7. Results, page 14: for some safety outcomes 'adjusted risk difference' is used, and for others it is 'risk difference' - is this distinction correct?

*Correct. Differences presented for the safety outcomes are unadjusted and should be described as a “risk difference” except for the proportion requiring respiratory support which was prespecified as a secondary outcome and thus has been adjusted for the randomisation stratification factors.*

**Reviewer #2:**

The manuscript presents a randomized controlled trial of physiologically based cord clamping (PBCC) for moderately preterm to term infants who have risk factors identified that merit presence of a pediatrician at delivery. The study adds important information on the feasibility and safety of PBCC in this group of infants who have not been extensively studied in previous trials, but who may realize significant benefit from initiation of ventilatory support with intact placental circulation. However, the results and conclusions of the study are constrained by limitations of heart rate data collection and the setting of high-resource referral centers with close monitoring, where most fetal distress was brief and not severe.

Abstract: The abstract clearly presents the aims, methods, and results.

-What did the researchers do and find? Instead of "infants assessed to require resuscitation following initial stimulation after birth", it might be more specific to say "infants assessed to require resuscitation following initial drying", as specific stimulation to breathe later in the sequence appeared to be part of the definition of resuscitation.

*Thank you for your comment. The word “drying” has been added to this bullet point. We typically tried tactile stimulation prior to the decision to randomize the babies. There was universal drying for all infants to put on ECG leads, which included drying of the head and face. If the infant wasn’t breathing, there was usually a further attempt to stimulate prior to a request for resus and subsequent randomization. Please see below for further discussion of this point.*

-What do these findings mean? The definition of "early heart rate" could be debated. Although acquisition with conventional ECG leads has been shown to be accurate, application of the leads delays the acquisition of data and important differences between groups may have been lost in the first 60 seconds.

*Thanks, the sentence now reads “For infants born at ≥32^+0^ weeks’ gestation requiring resuscitation, this trial did not show superiority for PBCC with regard to heart rate between 60-120 seconds after birth compared with ECC.”*

-The percentile charts provide valuable new information on an at-risk group; however, the limited number of subjects may mean that these provide early guidance on this group rather than best estimates of heart rate and oxygen saturation.

*Thanks for your comment. We have* *now removed the phrase “best estimates” in favour of “estimates”. Of note, the observational arm of our study has a similar number of infants ≥35 weeks who received DCC as the Dawson 2010 ADCFNE publication, widely used for benchmark values of HR and SpO_2_. We used ECG, which we feel is more accurate in the first 1-2 minutes after birth and our cohort includes infant born via caesarean section. We believe that this study presents more representative data for incorporation into clinical care. However, a larger data set that includes infants from other centres would improve the generalisability of the data, and have included this phrase in the Discussion (page 22):*

*“*However, a larger data set that includes infants from other centres would improve the generalisability of the data.”

Introduction: The introduction is very clear and addresses the main points concisely.

Methods: In the description of study procedures, it would be helpful to describe the sequence of events in very concrete terms. For example, instead of "infants were stimulated immediately after birth" would it be accurate to say that "infants were dried thoroughly immediately after birth" and that provided stimulation? "If the infant was non-vigorous and assessed to require ongoing resuscitation within 60 seconds of birth" leaves the reader wondering how randomization occurred in time for early clamping. Was the need for further resuscitation determined by cry/no cry after initial drying/stimulation? Did all infants judged to need further resuscitation receive specific stimulation to breathe (apart from initial drying) before beginning PPV?

*Thanks for this important comment. We did not standardize the type or amount of stimulation provided prior to randomization. After initial drying (that lasted ~10 seconds after birth), we typically attempted further stimulation before the decision to randomize (mean time to randomisation was 26 s after birth). This was to try and recruit infants who truly needed resuscitation. We are therefore less inclined to refer to these initial measures as thorough drying alone. The criteria for randomisation were intentionally not specified to reflect the variability in the degree of concern for the infant at birth that can depend upon fetal (e.g. gestational age), birth (e.g. shoulder dystocia), and clinician (e.g. experience) factors. These factors will influence how quickly after initial drying/stimulation the attending clinician may wish to commence resuscitation. As a result, infants were randomised if they remained apneic despite ongoing stimulation and were determined to need PPV by the attending clinician. We did not use the presence of cries as a criterion per se because we anticipated that some infants would have an initial quiet cry but subsequently become apneic. This complexity reflects the known difficulty of accurately assessing which infants are in primary versus secondary apnea and represents a pragmatic approach in the trial. To avoid bias, we therefore developed a method for rapid group allocation after assessment of need for resuscitation. We hope the following addition to the Methods section (page 9) succinctly addresses this comment:*

*“All infants were dried and stimulated immediately after birth. Infants were randomised if they remained apneic despite ongoing stimulation and were determined to need PPV by the attending clinician.”*

*“Randomised infants received specific interventions to breathe at the discretion of the attending clinician. These included ongoing vigorous stimulation and/or respiratory support.”*

-On page 9 the device used to provide respiratory support is described as a mobile resuscitator from GE Healthcare, USA, but on page 10 reference is made to the NeoPuff manometer (Fisher & Paykel).

*Thank you, this has been corrected.*

-The description of the analysis of video recording is excellent and emphasizes the clever use of technology to accomplish as much "blinding" as possible. It would be helpful to further describe the method for sampling heart rate at 10 s intervals.

*Thanks for the suggestion. The investigator at the birth edited the video of a randomized baby; cutting the video to start at the time of birth, cropping the picture to only capture the monitor screen, and removing the sound. The edited video was placed on the secure data drive and the investigator who was not at the birth extracted the data by watching it and recording the HR/SpO2 every 10 seconds. SB did most of the data collection at the Royal Women’s Hospital and DB did most of the data collection Monash, further reducing the risk of unblinding of either investigator. We have included an example of a blinded video in the Supplemental files.*

*“The video recording was cropped to include only the monitor screen and muted to sound (supplemental video) before being shared with an off-site researcher who was not in attendance at the birth. For all infants, HR and oxygen saturation data were extracted at 10 s intervals until 10 minutes after birth. ECG and SpO2 readings were only accepted if QRS complexes and plethysmograph waveforms respectively showed adequate signal quality.”*

Statistical analysis: What was the basis for selecting the heart rates and standard deviations for the PBCC and ECC groups? Not only were the heart rate ranges quite different from those observed, the spread between ranges was considerably less.

*We have added an explanation to the Methods. The expected HR of the ECC group was calculated from an audit of infants in a neonatal resuscitation video review quality improvement program who received PPV for apnea and had accurate HR data between 60-120 seconds. The expected HR for the PBCC group came from a small subset of non-vigorous babies in the the Baby DUCC feasibility study.*

*“The sample size calculation was based on data from an audit of infants requiring PPV for apnea and the previously published feasibility study.”*

Results: For the primary outcome, reporting heart rate to the nearest whole number would be sufficient and more realistic.

*Thank you. Heart rate is now reported in whole numbers in both the Results section and in Table 2.*

In the discussion of secondary outcomes, it would be clearer to define what was included in "resuscitation".

*Thank you. We have now added the relevant details:*

*“Following randomisation, more infants in the PBCC arm received any resuscitation (comprising stimulation to establish spontaneous breathing and/or respiratory support) compared to the ECC arm (98% vs 77%, adjusted risk difference 22.4%, 95% CI 11.3 - 34.6%).”*

The initial FiO2(s) for PPV in this mixed preterm/term group might also be specified.

*Thank you, this has now been specified in the Methods section (page 10):*

*“Infants received respiratory support using a mobile resuscitator (GE Healthcare, USA) set to pressures of 30/5 cmH_2_O and at 21% FiO_2_”.*

Was the percent of infants with HR <100 calculated on any heart rate < 100 or any heart rate sampled < 100?

*Any HR sampled <100. We evaluated the HR for quality of the ECG trace and extracted data at 10s intervals. There may have been a few cases where the HR dropped to <100 bpm between data points but the brevity of these drops in HR were renders them clinically less important.*

Similar to heart rate, time in seconds could be reported in whole numbers.

*Time in seconds is now reported in whole numbers in both the Results section and in Table 2.*

Discussion: The discussion is very thorough and brings out excellent points regarding the relatively "late" timing of early cord clamping and providing initial stimulation with cord intact. The discussion should also address the time needed for ECG signal acquisition, as this may have obscured differences in heart rate pattern between the two groups. Studies using dry-electrode ECG acquisition of heart rate recently have reported signal acquisition at 5-10 seconds (Bush JB et al ADCFN 2021, Bjorland PA et al. ADCFN 2021). The early contours of heart rate curves also appear to be different when comparing the randomized infants to those enrolled in the observational arm.

*Thanks, we did not have access to the dry electrodes (I asked) at the beginning of our study and we had to create a mechanism in which our monitoring equipment was sterile for use in c-sections. Plus, we wanted our technique to be easy to duplicate. Ethylene oxide sterilization is widely available and inexpensive for countries with a similar standard in care to Australia. Because infants randomised to ECC also had initial drying/stimulation with the cord intact until median [IQR] 37 s [27-51 s], there was unlikely to be differences in HR between the 2 groups at the very early timepoints.*

*The difference in the early HR curves is now expanded upon in the Discussion (page 19):*

*“Infants in the randomised trial had relatively lower HR in the first 3 minutes after birth compared to most infants in the observational arm. The difference in the early HR curves likely represents the relative hypoxia of infants in the randomised arms, reflecting appropriate randomisation of infants who required resuscitation versus infants in the observational arm who were less hypoxic at birth and therefore able to transition unassisted. This observation further supports the argument for a more nuanced appreciation of HR as an early marker of successful transition beyond the traditional dichotomisation around 100 bpm.^30^”*

The relatively long time from birth to establishment of ventilation in the ECC group may also be worth comment. The delay of 92 seconds is virtually identical to that reported from large observational studies in Tanzania. This emphasizes how difficult it is to initiate respiratory support quickly when umbilical cord clamping is performed before an infant is physically moved to a site for resuscitation.

*Thanks, we have now commented on the delay to get to the resuscitation platform in the Discussion (page 18).*

*“The approach of trying to initially stimulate spontaneous breathing prior to ECC and the need to subsequently move the infant to a separate resuscitation platform reflects current practice and explains the relatively long interval from birth to commencement of respiratory support.”*

Conclusion: The conclusion is appropriate, with the reservations expressed previously.

Figures and Tables: Figures and tables are valuable and complete.

References: Reference 27 spells out first names rather than surnames.

*This has been corrected, thanks.*

General: Minor point of usage and punctuation include:

-Physiologically-based cord clamping - editorial style might omit the hyphen

*Thanks for the suggestion, this has been a point of contention among the authors. Prof G Polglase is the first to have published this term in 2015. His preferred spelling is what we’ve been using.*

-Diaphragmatic herniae (page 8) - is this meant to be plural?

*Thanks, changed to singular.*

**Reviewer #3:**

Physiologically based cord clamping for infants ≥32+0 weeks gestation - a randomized clinical trial in reference percentiles for heart rate and oxygen saturation

Thank you for the opportunity to review Badurdeen and colleagues' manuscript, "Physiologically-based cord clamping for infants ≥32+0 weeks gestation - a randomized clinical trial in reference percentiles for heart rate and oxygen saturation".

The authors note that deferred cord clamping is now recommended as the standard for cord care. In addition, they note that many of these studies excluded infants who were thought to be at risk for needing resuscitation. The authors note that there may be improvement in infant transition if you avoid removal of the placental circulation by early or shortly delayed cord clamping prior to the establishment of breathing and overly early cord clamping may contribute to adverse outcomes. Keeping the infant connected to the placenta until breathing is established and holds promise as a low-cost and readily available means of improving newborn outcomes. In this trial, the authors test a physiologically based cord clamping (PBCC) procedure, which emphasizes the establishment of lung aeration, pulmonary gas exchange, and the increase in pulmonary blood flow prior to cord clamping.

In addition to exploring physiologically based cord clamping (PBCC), the authors realized that they had an exceptional opportunity to explore the transition of infants thought to be compromised but not needing resuscitation.

Current resuscitation guidelines recommend that infants receiving resuscitation achieve similar oxygen saturation levels to those observed in healthy infants. These targets are largely based on percentile charts published by some of these investigators in 2010 during the era of early cord clamping. The authors suggest a need to reevaluate the target physiologic parameters prescribed for infants receiving resuscitation.

For that reason, the authors sought two study goals: 1) to determine whether PBCC provides physiologic benefits versus ECC for infants requiring resuscitation at birth; and 2) to establish new normative percentile charts of heart rate and oxygen saturation for healthy infants who are at need of resuscitation prior to birth but who are vigorous and received deferred cord clamping.

This study is a two center, parallel arm, superiority randomized trial conducted in two Australian centers. Participants include infants born at ≥32 weeks gestation where a pediatrician has been requested to attend birth for the potential newborn compromise. This inclusion criteria is hard to understand in a more generalizable sense and should be specifically discussed either in the text or supplemental materials. Exclusion criteria are clearly discussed and are sensible regarding the possible compromise caused by undue delay in cord clamping.

*Thank you for your suggestion. The inclusion criteria have been expanded upon in the Methods (page 8):*

*“Infants born at ≥32+0 weeks’ gestation with a request for a paediatrician to attend the birth for potential newborn compromise were eligible (including unplanned/emergency caesarean sections or caesarean sections with breech presentation, instrumental assisted births, meconium stained liquor, multiples, infants <37+0 weeks gestation, or concern for fetal distress prior to birth).”*

Of note, the study had a unique enrollment and randomization procedure, one which is difficult to perform but excellently executed by this group. They received prospective written parental consent, which was deferred until after birth, when they randomized infants at <30 seconds of age if they required resuscitation. Randomization was stratified by study center and indication for pediatric attendance, including preterm birth, non-emergent birth ≥36 weeks gestation, and emergency birth ≥36 weeks gestation. Physician attendance was required for entry into the study. Infants were randomized either to physiologically based cord clamping, the establishment of an effective pulmonary gas exchange, either via positive pressure ventilation or effective, spontaneous breathing prior to cord clamping, or ECC immediate cord clamping, followed by resuscitation. The definition of early cord clamping is somewhat confusion as this group had at least a 30 second delay, based on the

randomization procedure itself.

*You are correct about 30 seconds. In our institutions, 60 seconds is considered DCC by the patients, midwives/OBs, or the neonatal teams. We have added a statement to the methods section under non-randomised infants:*

*“Non-randomised infants received a minimum of ≥120 seconds between birth and umbilical cord clamping. Of note, DCC at both study institutions is considered ≥60 seconds after birth in vigorous infants.”*

*For study enrolment we couldn’t realistically ask parents to consider less than standard of care for DCC. Therefore, the ECC group had cord clamping only after it was determined that the infant needed resuscitation, which is in contrast to historical trials of immediate cord clamping conducted prior to consensus recommendations on DCC for well infants. There is now a more expanded discussion of this point in the Discussion (page 18):*

*“Infants in the ECC group had cord clamping at a median (IQR) of 37 s (27-51 s) after birth, which is later than the historical approach of immediate cord clamping. This timeframe is consistent with current practice, where initial stimulation is provided with the cord intact, allowing many infants who will spontaneously cry the opportunity to have deferred cord clamping. These two factors may have contributed to short clamp-to-ventilation intervals and better than anticipated transition in infants receiving ECC. The approach of trying to initially stimulate spontaneous breathing prior to ECC and the need to subsequently move the infant to a separate resuscitation platform reflects current practice and explains the relatively long interval from birth to commencement of respiratory support.”*

A significant number of patients did not require resuscitation and were followed in an observational cohort. Data from these non-randomized infants who received ≥2 minutes of delayed cord clamping, per protocol, and remained vigorous after cord clamping until ≥10 minutes after birth were used to develop percentile ranges of oxygen saturation and heart rate.

508 infants were enrolled. Aside from a larger proportion of mothers in the physiologically based cord clamping arm having medical complications in pregnancy compared to the early cord clamping arm (43% to 25%), the groups were similar. Median gestation at birth was 39+5 weeks, mean time to randomization was 26 seconds after birth, and cord clamping occurred at a mean of 136 seconds in the PBCC group and 37 seconds in the ECC arms. Ultimately, the investigators noted no difference in heart rate overall or in the sub-groups based on gestational age and indication for delivery. More infants in the PBCC arm received resuscitation compared to the ECC arm (98% versus 77%, adjusted difference 22.4%, 95% CI, 11.3% to 34.6). A higher proportion of infants in the PBCC arm received positive pressure ventilation (48% to 25%) and respiratory support was initiated sooner than in the ECC infants. No differences in safety outcomes were reported. Routine hematocrits were not done and is a small deficiency of this study.

*Agreed, re: haematocrit. Most infants in the study were not admitted to the nursery and collection of haematocrit was not routine. We could have asked to use scavenged blood at the time of the newborn screen, but many babies in our institutions are on an early discharge pathway and could be home prior to collect of the newborn screen. Including details of additional blood tests during the consent process may have decreased consent rates. Finally, other studies have reported on haematocrit levels. We did not feel that our study would be properly powered to contribute substantially to this literature.*

The authors go on to describe the observational arm and the creation of percentile charts of heart rate and oxygen saturation. In the discussion, the authors state that physiologically based cord clamping led to similar results in heart rate and saturation compared to infants receiving early cord clamping. However, it seems naïve to state that safety outcomes and clinical outcomes were similar, as noted above. The authors include a lengthy discussion around creating the new percentile charts.

*With regard to the comment on safety and clinical outcomes, please see our response to the next point.*

This small but well-executed study is somewhat difficult to read. The authors combine two different exercises, both with very different methodologies. Some thought should be given to splitting out these two exercises (clearly the data from the observational trial being less important but of real interest). This would allow for much of the clinical data that is included in the first supplement to be added into an article and for authors to be able to look at many of the secondary outcomes that are associated with timing of cord clamping directly in manuscript itself. In addition, if anything, the study does not support the conclusion that physiologically based cord clamping results in similar effects. That may be true of the primary outcome (mean heart rate), and there seems to be no benefit and some potential harm from the delays in physiologically based cord clamping. More caution should be noted in the authors observations and conclusions.

*Thank you for your suggestions. We had carefully considered the option of splitting the results into two manuscripts. However, because infants in all study arms had identical inclusion/exclusion criteria and were derived from the same population, we felt that there were significant benefits for the reader to interpret the results from the RCT and observational cohorts alongside one another. We would be happy to move the safety outcomes table (Supplemental Table 1) to the main manuscript and seek the advice of the Editor.*

*We have reorganised the Discussion in line with the Editor’s suggestions and believe this significantly improves readability.*

*We have also added further qualifiers to our statements with regard to safety and secondary outcomes in line with the reviewer’s suggestions:*

*Under “What do these findings mean?” we have added the following statement:*

*“PBCC may provide other important benefits that we did not measure, including improved maternal-infant bonding and increased placental transfusion resulting in higher iron stores.”*

*In the limitations paragraph of the Discussion, we have added the following statement:*

*“The degree of confidence on safety outcomes being similar between the randomised groups is limited by the relatively small sample size.”*

*In the final paragraph of the Discussion, we have added the following statement:*

*“PBCC may yet be beneficial in infants who have more severe hypoxia and require more time and/or respiratory support after birth to establish pulmonary gas exchange, particularly if the utero-placental circulation is intact. This warrants further investigation alongside safety and long-term outcome data in a larger study population.”*
