# Supplementary material for: Physiologically based cord clamping for infants ≥32+0 weeks gestation: A randomised clinical trial and reference percentiles for heart rate and oxygen saturation for infants ≥35+0 weeks gestation
Source: PLoS Med. 2022 Jun 23;19(6):e1004029. doi: 10.1371/journal.pmed.1004029 (PMC9269938; doi:10.1371/journal.pmed.1004029)
Supplement: Attachment — Submitted filename: BDRCT_2nd response to reviewers.docx [file pmed.1004029.s006.docx]

**Response to editor and reviewers**

*Thank you for your thorough peer review of this manuscript, which has undoubtedly been strengthened. Please find below our responses to the requested edits.*

*Kind regards,*

*Shiraz Badurdeen, on behalf of the co-authors.*

**Requests from the editors:**

General comments:

Please refer to specific supplementary files e.g. S1 Table in the main text of your manuscript, rather than e.g. “Supplemental file”, “Supplemental Figures”, etc

*This has been revised as suggested.*

Title: Please revise your title to specify who the reference percentiles apply to e.g. “...reference percentiles for heart rate and oxygen saturation in infants ≥35+0 weeks’ gestation”

*This has been revised as suggested.*

Abstract Background: Please remove the sentence beginning “For infants not receiving resuscitation…”

*This sentence has been removed as suggested.*

Abstract Methods and Findings:

Please relocate sentences beginning “The trial was limited by the small number…” and “PBCC may provide other important…” to the end of the Methods and Findings section.

*This sentence has been relocated as suggested.*

Line 68 - Please revise to “with median (IQR) gestational age of…”

*This has been revised as suggested (line 69)*

Author Summary:

Please define PBCC at first use in your Author Summary

*This has been defined as suggested (line 118)*

Given the concerns of Reviewer 3 regarding potential harms, please temper assertions that “PBCC was safe and feasible” e.g. “Our findings suggest that PBCC is safe and feasible…”

*This has been revised as suggested (line 132)*

Methods:

Please provide the names of the institutional review boards that provided ethical approval and reference numbers in your Methods section (this can be moved from line 684).

*The ethics statement has been moved to the methods section (lines 218-221).*

Please add the following statement, or similar, to the Methods: "This study is reported as per the Consolidated Standards of Reporting Trials (CONSORT) guideline (S1 Checklist)."

*This statement has been added to the methods section (line 226).*

For the avoidance of issues relating to consent and copyright, it would be preferable if the accompanying videos could be uploaded to the Monash University Research Repository and cited, rather than uploaded as a supplementary file

*The supplementary videos have been added to the Monash University Research Repository and cited (reference 20).*

Discussion:

Given the concerns of Reviewer 3 regarding potential harms, please temper assertions that “PBCC was safe and feasible” e.g. “Our findings suggest that PBCC is safe and feasible…”

*This has been revised as suggested (line 644).*

Figures:

Please define the abbreviations in Figure 1 e.g. MCDA

*MCDA has now been defined in the figure legend (line 995).*

References:

Please ensure that journal name abbreviations match those found in the National Center for Biotechnology Information (NCBI) databases (http://www.ncbi.nlm.nih.gov/nlmcatalog/journals), and are appropriately formatted and capitalised. E.g ref 1 should be ‘Lancet’ rather than ‘The Lancet. Lancet Publishing Group’; ref 17 should be ‘J Biomed Inform’ instead of ‘Journal of Biomedical Informatics. Academic Press Inc.’, etc

*Journal names have been updated to match the NCBI database.*

Supplementary files:

Please name individual supplementary files to contain an "S" and number, as per https://journals.plos.org/plosmedicine/s/supporting-information

*Supplementary files have been renamed as suggested.*

Comments from Reviewers:

Reviewer #1: Thank you to the authors for addressing my previous comments well. I have no further issues to raise.

Reviewer #2: Thanks to the authors for the many improvements in response to reviewer comments; these have strengthened the manuscript significantly. With very minor adjustments to make language consistent through the manuscript, the revision is acceptable for publication.

The explanation of the approach to initial care at birth prior to randomization (drying and stimulation) is a significant part of the trial that deserves emphasis in the abstract and conclusion. Although many centers now are taking this approach, it differs from the original definition of early cord clamping, which often occurred within 10-15 seconds after birth.

Wording of the conclusion in the abstract differs slightly from that in the body of the manuscript ("limited benefits" in abstract vs."does not provide additional benefit" in line 454). The latter wording most accurately reflects the data, but the conditions of ECC performed after initial drying and stimulation deserve mention in both places. (The findings suggest that for infants>=32+0 weeks gestation who respond to brief, effective resuscitation at closely monitored births, PBCC does not demonstrate benefit over ECC performed after initial drying and stimulation in terms of key physiological markers of transition.)

*This has been revised as suggested (lines 79-80).*

If post-partum hemorrhage was the primary safety outcome, it may be pertinent to describe it as such in the abstract (e.g. Among 31 secondary outcomes, the safety outcome of post-partum haemorrhage......line 63).

*As PPH was not prespecified as a primary safety outcome, we would prefer to not describe it as such in the abstract. As per the CONSORT extension for abstracts, we consider it important to report the safety outcome that will be of greatest interest to readers. We appreciate the reviewer’s previous suggestion to report this outcome alongside the total number of secondary outcomes. This addition was made in the last revision of the manuscript.*

In line 268, the concentration of oxygen should be expressed as a fraction (0.21 FiO2) or a percentage (21% oxygen).

*This has been revised as suggested (line 295).*

The definition of deferred clamping in the introduction (line 187) as >30-60 seconds creates some confusion with ECC as practiced in the study (27-51 seconds) and the standard of care for deferred clamping at the study sites (> 60 seconds).

*This has been revised as suggested. DCC is now referred to as >60 seconds (line 204).*
